# A potential source for cellulolytic enzyme discovery and environmental aspects revealed through metagenomics of Brazilian mangroves

**DOI:** 10.1186/2191-0855-3-65

**Published:** 2013-10-26

**Authors:** Claudia Elizabeth Thompson, Walter Orlando Beys-da-Silva, Lucélia Santi, Markus Berger, Marilene Henning Vainstein, Jorge Almeida Guima rães, Ana Tereza Ribeiro Vasconcelos

**Affiliations:** 1 Laboratório Nacional de Computação Científica, Rio de Janeiro 25651070, Brazil; 2 Centro de Biotecnologia, Universidade Federal do Rio Grande do Sul, Rio Grande do Sul 91501070, Brazil

**Keywords:** Brazilian mangrove, Cellulase, Metagenomics, Biomass decomposition, Bacterial diversity

## Abstract

The mangroves are among the most productive and biologically important environments. The possible presence of cellulolytic enzymes and microorganisms useful for biomass degradation as well as taxonomic and functional aspects of two Brazilian mangroves were evaluated using cultivation and metagenomic approaches. From a total of 296 microorganisms with visual differences in colony morphology and growth (including bacteria, yeast and filamentous fungus), 179 (60.5%) and 117 (39.5%) were isolated from the Rio de Janeiro (RJ) and Bahia (BA) samples, respectively. RJ metagenome showed the higher number of microbial isolates, which is consistent with its most conserved state and higher diversity. The metagenomic sequencing data showed similar predominant bacterial phyla in the BA and RJ mangroves with an abundance of Proteobacteria (57.8% and 44.6%), Firmicutes (11% and 12.3%) and Actinobacteria (8.4% and 7.5%). A higher number of enzymes involved in the degradation of polycyclic aromatic compounds were found in the BA mangrove. Specific sequences involved in the cellulolytic degradation, belonging to cellulases, hemicellulases, carbohydrate binding domains, dockerins and cohesins were identified, and it was possible to isolate cultivable fungi and bacteria related to biomass decomposition and with potential applications for the production of biofuels. These results showed that the mangroves possess all fundamental molecular tools required for building the cellulosome, which is required for the efficient degradation of cellulose material and sugar release.

## Introduction

Mangroves are coastal ecosystems that are found in tropical and subtropical regions and that cover between 60 and 75% of the world’s coasts (Holguin et al. 2001). These ecosystems have unique characteristics, including brackish water, particular sediments (muddy soil), and a specific population of animals and plants. They represent a complex and dynamic ecosystem that varies in terms of its salinity, water levels and nutrient availability during the seasons (Gomes et al. 2008; Gonzalez-Acosta et al. 2006). Mangroves are among the most productive and biologically important environments (Giri et al. 2011). They play a critical role in water filtering and establishing and expanding the coastal line, and they are of fundamental importance in maintaining the food chain and carbon cycle for human, coastal and marine communities (Giri et al. 2011; Gomes et al. 2008).

Mangrove forests are often located in urban areas touched by constant anthropogenic activity, which is thought to be the major cause of coastal wetland deterioration. Brazil is the third most mangrove-rich country, with about 7% of the world’s total area of mangroves (Giri et al. 2011). However, these areas are under constant flux due to both natural and anthropogenic forces. The mangrove forests are under immense pressure from clear-cutting, land-use changes, hydrological alterations, chemical spills and climate changes. Due to their singular characteristics and nutrient availability, mangroves have a remarkable influence on microbial communities, and their activities are responsible for several processes in organic matter degradation and most of the carbon flow in their sediments (Holguin et al. 2001).

Importantly, mangrove forests are believed to play a role in the uptake and preservation of polycyclic aromatic hydrocarbons (PAHs). PAHs are contaminants with both known and suspected carcinogenic, toxic, and mutagenic properties. They enter the aquatic environments through atmospheric deposition, municipal effluents, industrial wastewater, and oil spillage (Zhou et al. 2008). The mangroves are exposed to anthropogenic PAH contamination from tidal water, river water and land-based sources. Consequently, they supply organic matter to the coastal waters and influence global biogeochemical nutrient cycling (Sun et al. 2012).

The adaptation of bacterial species to the mangrove ecosystem indicates a potential source of biotechnological resources. The mangroves could provide a rich resource for the discovery of new bacterial and fungal species that produce enzymes and molecules that could be used for human life, agriculture, industry and bioremediation (Dourado et al. 2012; Dias et al. 2009). The ability to convert lignocellulosic substrates in nutrients of low complexity is crucial for the carbon cycle and microbial survivability, especially in environments with large amounts of these substrates (Medie et al. 2012). Lignocellulosic materials are very difficult to degrade due to their dense and compact structural features, which are related to the protection of plants against microbial attack (Kumar et al. 2008). Microorganisms that specialize in vegetable biomass degradation might be found in specific environments, such as the mangrove ecosystem, where such biomass is abundant. These microorganisms, which have an enormous ecological relevance, may also have a biotechnological use in the production of biofuels.

Unlike other strategies used for novel enzyme and microorganism identification, metagenomic analysis has clear benefits as a powerful alternative to culture-dependent methods. High-throughput pyrosequencing is a new tool for the study of microbial ecology and can reveal the taxonomic diversity of specific environments at a high resolution (Zhu et al. 2011). It is becoming the most powerful tool for studying uncultured microbes and novel enzymes with biotechnological potential (Duan et al. 2010; Brulc et al. 2009). A metagenomic pyrosequencing-based approach for the isolation of cultivable microorganisms could accelerate the assessment of new enzymes and molecules and their possible application in biotechnological purposes. This approach may also be useful in the study of biodiversity and provide evidence about environmental interference.

The objective of this work was to evaluate the possible presence of cellulolytic enzymes and microorganisms useful for biomass degradation as well as taxonomic and functional aspects of two Brazilian mangroves. Using a metagenomic approach, the specific sequences and microorganisms involved in cellulolytic degradation were identified, and several cellulolytic microorganisms were isolated from the mangrove samples. The data provided here demonstrate the biotechnological potential of this unique but endangered environment.

## Material and methods

### Sample collection and DNA extraction

Samples were collected from two different Brazilian mangroves: sample RJ was collected from a preserved mangrove in the Rio de Janeiro state Ilha Grande during October of 2010 (23° 10' 11.852" S, 44° 17' 0.2" W), and sample BA was collected from a peri-urban mangrove area with anthropogenic action in the vicinity of the Bahia state Porto Seguro during November of 2010 (16° 35' 17.225" S, 39° 5' 30.642" W) (Additional file 1: Figure S1). In relation to the BA sample, no specific permissions were required according to the Brazilian government rules (resolution n. 21, August 31, 2006 - Ministry of Environment). The RJ sample was collected in accordance with the Brazilian law (IN 154/2007 IBAMA, Brazilian Institute of Environment and Renewable Natural Resources) and we confirm that the field studies did not involve endangered or protected species.

The samples (50 g) were collected from the soil surface (0–10 cm) using sterilized spatulas and sterilized 50 mL plastic tubes. The samples were maintained on ice during transportation until processing. Some physical-chemical and environmental characteristics of the samples were determined, such as their pH, temperature, and salinity. The samples collected were manually shaken for 2 minutes in 100 mL sterile Erlenmeyer flasks and 10 g were taken for DNA extraction. DNA was extracted from 10 g of each soil replicate using the PowerMax™ Soil DNA Isolation Kit (MoBio Laboratories, Inc., Carlsbad, CA, USA) according to the manufacturer’s recommendations. The quantity and quality of the resulting DNA were verified with a NanoDrop (Thermo Scientific, Wilmington, DE, USA) spectrophotometer at an absorbance ratio of 260/280 nm and 260/230 nm and were confirmed by electrophoresis on a 1% agarose gel. The DNA samples were then diluted to a concentration of 50 ng/μL.

### The isolation of cellulolytic microorganisms

Approximately 5 g of soil collected from both mangroves were inoculated in 200 mL of minimal medium MM (peptone 0.3%, K_2_HPO_4_ 0.05%, MgSO_4_ 0.05%) containing sugar cane bagasse 3% as the carbon source. The culture flasks were shaken for 5 min and maintained without agitation during 3 days at 28°C; the flasks were then incubated for 10 days at 28°C and 100 rpm. Samples of 50 μL each were collected in two-day intervals and cultured on Petri dish plates containing four different types of media: minimal medium with sugar cane bagasse 1% (M1), minimal medium with carboxymethyl cellulose (CMC) 1% (M2), Luria-Bertani (LB) medium (M3), and Sabouraud (M4) medium. Microorganisms with different colony morphologies were isolated and used to generate pure cultures, which were subsequently maintained at 4°C.

The cellulolytic ability of each isolate was tested by inoculation into 3 mL tubes of minimal medium with CMC plus sugarcane bagasse, pinus (*Pinus elliottii*) powder, or medium-density fiberboard (MDF) powder at 1%, as the main carbon source. After incubation for 5 days at 28°C and 150 rpm, the isolates that grew in at least in two of these substrates were also tested in a CMC-Congo red plate assay (Teather et al. 1982).

### DNA pyrosequencing and sequence processing

The preparation of two libraries was accomplished according to instructions from the Rapid Library Preparation Method Manual - GS FLX Titanium Series (454-Roche). The first library was made from 500 ng of DNA extracted from the RJ mangrove soil sample and another from 500 ng of DNA extracted from the BA mangrove soil sample. The titration, emulsion PCR, and sequencing steps were performed according to the manufacturer’s instructions. A two-region 454 sequencing run was performed on a 70x75 PicoTiterPlate (PTP) using the Genome Sequencer FLX System (Roche). Each region was loaded with one of the library preparations.

The artificially replicated sequences that were an artifact of the 454-based pyrosequencing technique were identified and eliminated using the Replicates software (Gomes-Alvarez et al. 2009). The remaining reads were further filtered with the LUCY program to remove short sequences (less than 180 bp) and sequences with a phred quality ≤ 20 (Chou and Holmes 2001).

The Newbler Assembler software version 2.5.3 was used to perform the assembly procedures, with a “-rip” flag that allows for the output of each read in a contig. Reads identified by the GS De Novo Assembler as problematic (Partial, Repeat, Outlier, TooShort) or with High-Quality Discrepancies (HQD) were filtered from the dataset. Subsequently, a new cycle of assembling and filtering was performed. These steps were repeated until the problematic and high-quality discrepancy reads were decreased below the cutoff threshold of 1% of the total number of reads filtered out at the first assembly step.

A total of 621,748 and 647,534 reads were produced by the 454-Titanium pyrosequencer after raw data processing for the BA and the RJ mangroves, respectively. The Replicates tool identified and removed 58,121 (BA) and 92,644 (RJ) replicate reads. From the remaining 563,627 and 554,890 reads, the LUCY tool removed another 60,608 and 60,689 low-quality reads, such that 503,019 (BA) and 494,201 (RJ) sequences were deemed appropriate for further taxonomic and functional categorization (Table 1). As a result of the assembly, 419,591 and 481,593 reads remained as singletons, while 72 and 29 contigs ≥ 500 bp were formed for BA and RJ, respectively. The average contig size was 23,788 bp for the RJ and 1,002 bp for the BA metagenomes.

**Table 1:** Summary of metagenomic data obtained from the mangrove microbiomes

**Parameters**	**Rio**	**Bahia**
**MG-RAST ID**	44852183	44852193
**No. of sequences**	494,201	503,019
**Avg. length (bp)**	327 ± 110*	328 ± 111*
**Total length (bp)**	161,854,245	165,387,021
**Predicted proteins**^**†**^	488,630	450,087
**Assigned reads**	242,868	261,482
**LCA**^**¥**^		
*Bacteria* (%)	206,733 (94,3)	273,078 (97,36)
*Archaea* (%)	10,041 (4,6)	4,623 (1,65)
*Eukarya* (%)	1,559 (0,7)	1,018 (0,36)
*Viruses* (%)	297 (0,1)	29 (0,01)
Unclassified (%)	649 (0,3)	1,740 (0,62)
**MEGAN**		
**No. of sequences**	494,201	503,019
**Assigned reads**	293,374	340,668
*Bacteria* (%)	252,395 (93,6)	313,633 (97,3)
*Archaea* (%)	13,173 (4,88)	5,686 (1,76)
*Eukarya* (%)	3,295 (1,22)	2,805 (0,87)
*Viruses* (%)	554 (0,2)	57 (0,01)
Unclassified (%)	269 (0,1)	182 (0,06)

*After duplicate removal, splitting and trimming of sequence reads.

^†^Predicted protein coding regions assigned an annotation using at least one of protein databases (M5NR) in MG-RAST server.

^**¥**^Lowest common ancestor (LCA) using 1e-5 cutoff, 60% minimum identity, and a minimum alignment lenght cutoff of 15.

Metagenome data reported in this paper have been deposited into the GenBank database (PRJNA186597) and on the MG-RAST server (4485218.3, 4485219.3, 4485981.3, and 4485982.3).

### Taxonomic distribution analysis

The taxonomic distribution of each read was performed with the MEGAN4 (Huson et al. 2011) software, which compares the given reads against a database of reference sequences by performing a search using the BLASTX algorithm (Altschul et al. 1997) against the NCBI-NR protein database. The MG-RAST server (Meyer et al. 2008) was also used for the taxonomic analysis. The 16S rRNA identification was accomplished using the Meta_RNA software (Huang et al. 2009). The sequences were subsequently classified with RDP Classifier software (Wang et al. 2007) based on the RDP Database (Cole et al. 2009).

### Functional metagenomic analysis

The MEGAN4 software was used to perform a functional analysis using the SEED (Overbeek et al. 2005) and KEGG (Kanehisa et al. 2012) databases. Each read was related to its SEED functional role using the best BLAST score to protein sequences without known functional roles. A similar procedure was used to match each read to a KEGG orthology (KO) accession number. To study specific metabolic pathways, the BA and RJ metagenomes were compared using 1e-05 as the maximum e-value cutoff, with a minimum identity of 60% and a minimum alignment length of 15. A functional comparison against the public metagenomes was also performed on the MG-RAST server.

Carbohydrate-active enzyme domains assigned by the CAZy database (Cantarel et al. 2009) were searched for in the predicted protein sequences (Rho et al. 2010; Zhu et al. 2010) using HMMER 3.0 software (Finn et al. 2011) and the Pfam 26.0 database (Punta el al. 2012), with a cutoff of E ≤ 1e-4.

All contig sequences were analyzed and functionally annotated using the System for Automated Bacterial Integrated Annotation (SABIA) (Almeida et al. 2004). According to the automatic annotation criteria, a given ORF was considered “valid” if it had BlastP hits on the KEGG, NCBI-nr or UniProtKB/Swiss-Prot databases, if it had subject and query coverage of ≥ 60%, and if it had positives of ≥ 60%. ORFs that had no BlastP hits on the NCBI-nr, KEGG, UniProtKB/Swiss-Prot, TCDB or Interpro databases or that were excluded by the above criteria were considered “hypothetical”.

### Comparative metagenomic analysis

The mangrove samples were compared with other metagenomes using the SEED subsystem content as a comparative metric (Suen et al. 2010 Tringe et al. 2005). Metagenomes from host-associated, water, and soil samples were evaluated using the MG-RAST server. The parameters for inclusion were a maximum e-value cutoff of 1e-05, a minimum identity of 60%, and a minimum alignment length of 15.

Trends in the abundance of the SEED subsystem were examined using Principal Component Analysis (PCA) and hierarchical clustering (Willner et al. 2009). The PCA method is a reduction/ordination technique that clusters the samples based on variations extracted from their normalized abundance profiles (Meyer et al. 2008). This analysis was performed on normalized data, using the bray-curtis dissimilarity.

### Statistical analyses

The MG-RAST taxonomic and functional profiles were analyzed using the Statistical Analyses of Metagenomic Profiles (STAMP) software (Parks and Beiko 2010) to detect biologically relevant differences in the relative proportions of the classified sequences. The two-sided Fisher’s exact test with Storey’s FDR method for multiple test correction was employed to analyze the data sets. All unclassified reads were removed from analyses. The most important taxa were filtered according to their q-values (0.01), and only those categories with more than a 2-fold ratio between the proportions or with difference between the proportions of at least 5% were used.

Statistical tests on the taxonomic data were also performed with MEGAN. The BA and RJ counts were normalized to produce data sets of 100,000 reads. Subsequently, MEGAN was used to apply a directed homogeneity test in order to highlight the significant differences in the mangrove comparisons. The highlighting thickness is logarithmically proportional to its significance; that is, the thickness is an integer value of 2log *x* when *P* = 1.0e^x^ (Mitra et al. 2009). Multiple testing correction analysis was not applied, and all unassigned reads were ignored.

## Results

### Sample description

Two different mangrove locations with distinct features were chosen: one in the Southeast (RJ) and the other in the Northeast (BA) of the country. The RJ mangrove is located in the city of Angra dos Reis, in Rio de Janeiro state, on a natural reserve island called Ilha Grande. Ilha Grande was created by the Brazilian government on August 25, 1978, and is considered to be highly preserved. It is 3,600 ha in size and contains both mangroves and other coastal ecosystems, including the Atlantic rainforest of subtropical southeast Brazil. The BA mangrove is located in the city of Porto Seguro, in the Trancoso district of the Bahia state, in a tropical region of the continent. It also contains both coastal and restinga ecosystems (Additional file 1: Figure S1). Despite its allocation in a hard-to-access site, the area is close to several small rural properties and a few luxury beach hotels and resorts. Consequently, this area is now increasingly threatened by urban development. Therefore, the BA mangrove is considered to be at a medium grade of degradation (Gomes et al. 2008). Although these two mangrove systems differ in terms of their location (island and continent), anthropic action and preservation state, they are similar in terms of their physical-chemical parameters. They both have a humid climate with average temperatures of 24°C and average salinity levels of 1.5%. The RJ mangrove has a neutral pH of 7.0, while the BA mangrove has a slightly acidic pH of 6.5. The samples were collected from the soil surface and the intertidal zone to access different soil layers along with their associated microorganisms.

### The isolation and selection of cultivable microorganisms

**Figure 1:**
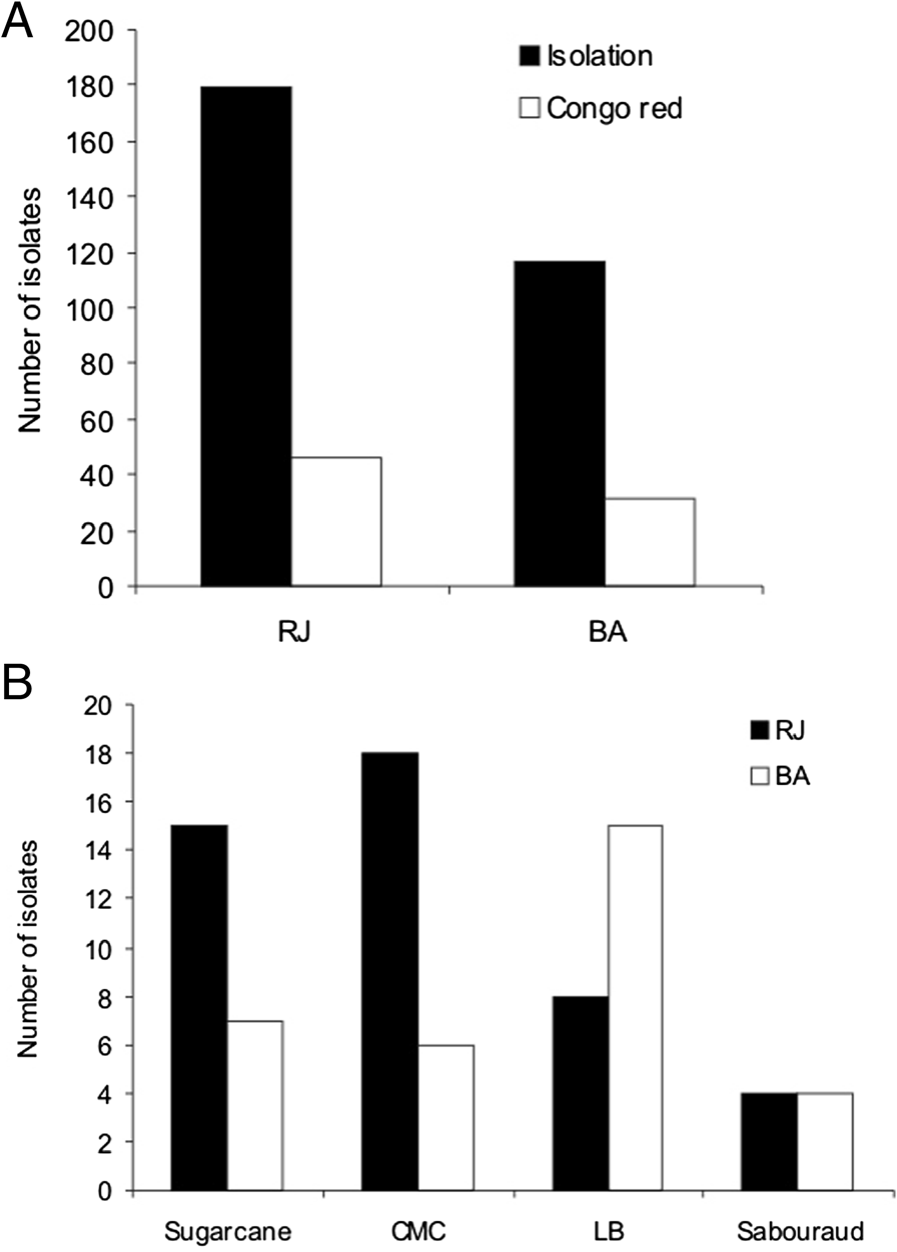
**Cellulolytic microorganisms isolated from Brazilian mangroves. (A)** Total number of microbial isolates and positives for CMC-Congo red plate assay. **(B)** Number of microbial isolates corresponding to each culture medium in the first step of isolation.

Samples from the RJ and BA mangroves were used to isolate cultivable microorganisms in four different media. From a total of 296 microorganisms with visual differences in colony morphology and growth (including bacteria, yeast and filamentous fungus), 179 (60.5%) and 117 (39.5%) were isolated from the RJ and BA samples, respectively (Figure 1A).

To determine the ability of these isolates to degrade cellulosic compounds, all microorganisms were grown in a CMC-congo red assay. Here, 77 microorganisms were found to be positive, and 58.4% of these were isolated from the RJ mangrove. In addition, 73% of the CMC-congo red positive microorganisms were initially isolated from mangrove samples through media containing cellulosic compounds (sugarcane bagasse or CMC) (Figure 1B). Twenty-one filamentous fungi with cellulolytic potential were isolated.

### The biodiversity and general characteristics of the mangrove metagenome

A large-scale metagenomic analysis was used to profile the microbiota of two Brazilian mangroves and to identify their functional attributes. A total of 503,019 and 494,201 reads from BA and RJ, respectively, were submitted for taxonomic and functional analysis. After applying quality control measures and removing any artificial duplicate reads (ADRs) on the MG-RAST server, the 491,224 (98.7%) reads from the BA library produced a total of 450,087 predicted protein-coding regions. Similarly, a total of 488,630 protein-coding regions were predicted from the RJ mangrove metagenome (Table 1). Of these sequences, 772 (BA) and 457 (RJ) were identified as 16S rDNAs using the MG-RAST server against RDP. This result corresponds to 0.15% and 0.09% of the total sequences obtained from the BA and RJ samples, respectively.

For the BA metagenome, 261,482 (58.1%) of the 450,087 predicted proteins were assigned an annotation, and 188,605 (41.9%) had no significant similarity to any protein in the databases and were therefore considered orphan sequences. In total, 243,812 (93.2%) sequences were functionally categorized. In the RJ sample, 242,868 (49.7%) sequences were assigned an annotation, and 245,762 (50.3%) were found to have no significant similarities to anything in the protein databases. Of the annotated sequences, 224,512 (92.4%) were assigned to functional categories. The average read length for the BA sample was 328 ± 111 bp, with a mean GC content of 54 ± 10%, and the average read length for the RJ sample was 327 ± 110 bp with a mean GC content of 55 ± 10% (Table 1).

The MEGAN results assigned annotations to 340,668 reads from the BA mangrove, which corresponds to 67.7% of the total reads, 162,169 (32.2%) of which are orphan sequences. For the RJ sample, 293,374 (59.3%) reads had significant similarities to hits within the protein databases, whereas 200,458 (40.5%) were unassigned reads. The algorithm implemented by MEGAN assigned more sequences when compared to MG-RAST, and it was able to identify a higher number of sequences related to *Bacteria*, *Archaea*, *Eukarya*, and *Viruses* (Table 1).

The alpha diversity was calculated for both samples, and the results indicated that the RJ mangrove (alpha-diversity = 974.385 species) is more diverse than the BA mangrove (alpha-diversity = 847.721 species). The index of alpha-diversity summarizes the diversity of organisms in a sample and is estimated according to the distribution of species-level annotations.

### The analysis of mangrove-associated microbiota

**Figure 2:**
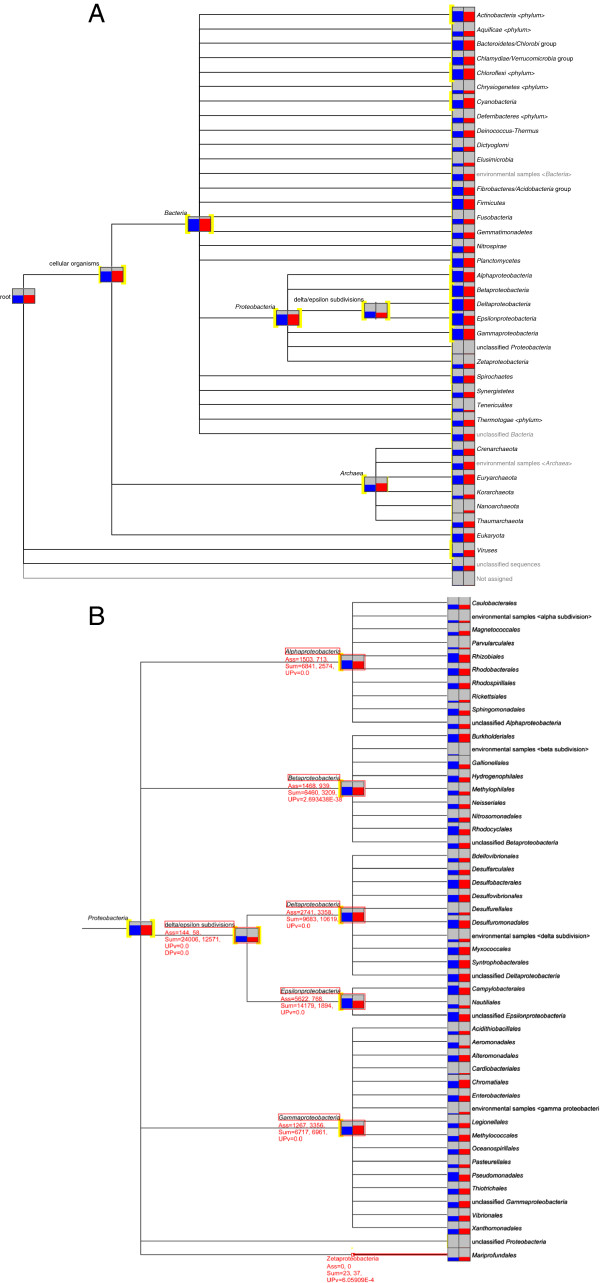
**Analysis of the composition of mangrove microbial communities, showing the taxonomic diversity of metagenomic sequences, computed by MEGAN based on a BLASTX using an e-value cutoff of 1e-5 comparison for Bahia (blue) e Rio de Janeiro (red). (A) ***Bacteria*, *Archaea*, *Eukaryota*, and *Viruses*. **(B) ***Proteobacteria*. The assigned and summarized reads for *Alphaproteobacteria*, *Betaproteobacteria*, *Deltaproteobacteria*, *Epsilonproteobacteria*, and *Zetaproteobacteria* nodes are displayed. Yellow highlighting on the left side of a node indicates that the up-test of directed homogeneity test showed a significant difference. The thickness of the highlighting is logarithmically proportional to the significance. The size of the bars is scaled logarithmically to represent the number of reads assigned to each taxon. The UPv labels indicate the P-values associated with the up parts of the directed homogeneity test.

At the domain level, *Bacteria* were more abundant than *Archaea* in the two mangrove samples (Additional file 2: Figure S2, Table 1). The MG-RAST analysis indicated that the percentage of sequences affiliated with each taxa were similar in the BA and RJ mangroves, with an abundance of *Proteobacteria* (57.8% and 44.6%), *Firmicutes* (11% and 12.3%), *Actinobacteria* (8.4% and 7.5%), *Bacteroidetes* (4.1% and 4.3%), *Chloroflexi* (3.2% and 6.4%), *Cyanobacteria* (2.2% and 5.8%), *Euryarchaeota* (2.2% and 4.5%), and *Planctomycetes* (1.6% and 1.9%). The statistical test applied by MEGAN revealed that the proportional difference in reads assigned to *Chloroflexi*, *Cyanobacteria*, *Planctomycetes*, *Proteobacteria*, and *Chlamydiae*/*Verrucomicrobia* was highly significant between the BA and RJ mangroves, with the RJ sample having a higher number of reads assigned to those taxa. Moreover, the *Actinobacteria*, *Fibrobacteres*/*Acidobacteria*, and *Firmicutes* groups were significantly more highly represented in the BA mangrove (Figure 2A).

In terms of the phylogenetic classification within *Proteobacteria* obtained via MG-RAST, the most frequent class detected was *Gammaproteobacteria* (13.5% in BA and 13.6% in RJ), followed by *Epsilonproteobacteria* (11.6%), *Alphaproteobacteria* (11.5%), *Deltaproteobacteria* (10.9%), and *Betaproteobacteria* (9.9%) in the BA sample, and followed by *Deltaproteobacteria* (12.5%), *Betaproteobacteria* (8.2%), *Alphaproteobacteria* (7.9%), and *Epsilonproteobacteria* (2.1%) in the RJ sample. The statistical analyses from MEGAN indicated that the BA mangrove had significantly more reads in *Alphaproteobacteria*, *Betaproteobacteria*, and *Epsilonproteobacteria*, whereas the RJ mangrove had significantly more reads in *Gammaproteobacteria*, *Deltaproteobacteria*, and *Zetaproteobacteria* (Figure 2B).

At the genus level, *Pseudomonas* (4.3%), *Campylobacter* (2.9%), and *Geobacter* (2.2%) were found to be the main bacterial groups in the BA sample, as measured according to the MG-RAST results. In contrast, *Geobacter* (2.2%), *Roseiflexus* (1.9%), and *Burkholderia* (1.8%) were found to be the main bacterial groups in the RJ sample.

The BA and RJ mangroves were statistically different in terms of their *Archaea* abundance, with the RJ sample having a higher abundance (Figure 2A). The majority of Archaeal EGTs corresponded to methanogenic classes, with the largest proportion corresponding to the *Euryarchaeota* (Figure 2A). The genomes of the most representative strains from each mangrove are quite different (Table 2). Anaerobic bacteria are more heavily represented in the RJ mangrove, several of which belong to the *Deltaproteobacteria* genus. In contrast, the BA mangrove has several reads from *Actinobacteria*, which are not heavily represented in the RJ mangrove samples. Moreover, some Euryarchaeota genomes were identified only in the RJ sample (Table 2).

**Table 2:** Sequenced genomes with most hits to the mangrove metagenomes

**RJ Mangrove**								
**Genome**	**Kingdom/Group**	**Gram Strain**	**Oxygen requeriment**	**Motility**	**Pathogenic**	**Habitat**	**Salinity**	**Number of reads**
*Syntrophobacter fumaroxidans* MPOB	*Bacteria*/*Deltaproteobacteria*	Negative	Anaerobic	No	No	Aquatic		1,339
*Desulfatibacillum alkenivorans* AK-01	*Bacteria*/*Deltaproteobacteria*	Negative	Anaerobic	No	No	Aquatic		1,007
*Syntrophus aciditrophicus* SB	*Bacteria*/*Deltaproteobacteria*	Negative	Anaerobic	No		Multiple		584
*Thioalkalivibrio sulfidophilus* HL-EbGr7	*Bacteria*/*Gammaproteobacteria*	Negative	Aerobic	Yes	No	Specialized	Moderate halophilic	472
*Roseiflexus* sp. RS-1	*Bacteria*/*Chloroflexi*	Negative	Facultative	Yes		Specialized		421
*Gemmatimonas aurantiaca* T-27	*Bacteria*	Negative	Aerobic	Yes	No			374
*Opitutus terrae* PB90-1	*Bacteria*		Anaerobic	Yes	No	Aquatic		290
*Thermomicrobium roseum* DSM 5159	*Bacteria*/*Chloroflexi*	Negative	Aerobic	No	No	Specialized		224
*Geobacter uraniireducens* Rf4	*Bacteria*/*Deltaproteobacteria*	Negative	Microaerophilic			Multiple		222
*Thiobacillus denitrificans* ATCC 25259	*Bacteria*/*Betaproteobacteria*	Negative	Facultative	Yes	No	Multiple		214
*Methanosarcina acetivorans* C2A	*Archaea*/*Euryarchaeota*		Anaerobic	No	No	Aquatic		179
*Methanoculleus marisnigri* JR1	*Archaea*/*Euryarchaeota*	Negative	Anaerobic	Yes	No	Aquatic		170
BA Mangrove								
Genome	Kingdom/Group	Gram Strain	Oxygen requeriment	Motility	Pathogenic	Habitat	Salinity	Number of reads
*Thiobacillus denitrificans* ATCC 25259	*Bacteria*/*Betaproteobacteria*	Negative	Facultative	Yes	No	Multiple		984
*Syntrophobacter fumaroxidans* MPOB	*Bacteria*/*Deltaproteobacteria*	Negative	Anaerobic	No	No	Aquatic		911
*Desulfatibacillum alkenivorans* AK-01	*Bacteria*/*Deltaproteobacteria*	Negative	Anaerobic	No	No	Aquatic		771
*Syntrophus aciditrophicus* SB	*Bacteria*/*Deltaproteobacteria*	Negative	Anaerobic	No		Multiple		492
*Gemmatimonas aurantiaca* T-27	*Bacteria*	Negative	Aerobic	Yes	No			326
*Opitutus terrae* PB90-1	*Bacteria*	Negative	Anaerobic	Yes	No	Aquatic		250
*Geobacter uraniireducens* Rf4	*Bacteria*/*Deltaproteobacteria*	Negative	Microaerophilic			Multiple		230
*Roseiflexus* sp. RS-1	*Bacteria*/*Chloroflexi*	Negative	Facultative	Yes		Specialized		220
*Rubrobacter xylanophilus* DSM 9941	*Bacteria*/*Actinobacteria*	Positive	Aerobic	No	No	Specialized		211
*Nocardioides* sp. JS614	*Bacteria*/*Actinobacteria*	Positive	Aerobic	No	No	Terrestrial		157
*Thioalkalivibrio sulfidophilus* HL-EbGr7	*Bacteria*/*Gammaproteobacteria*	Negative	Aerobic	Yes	No	Specialized		151

According to the MEGAN results, the majority of the eukaryotic EGTs (40.1 and 37.2% for BA and RJ, respectively) were more similar to the *Opisthokonta*, *Stramenopiles* (21.8 and 32.2%), and *Viridiplantae* (24.3 and 18.7%, respectively). However, there was no significant difference between the mangroves in terms of either their eukaryotic or their viral sequences.

### Functional metagenomic analyses

**Figure 3:**
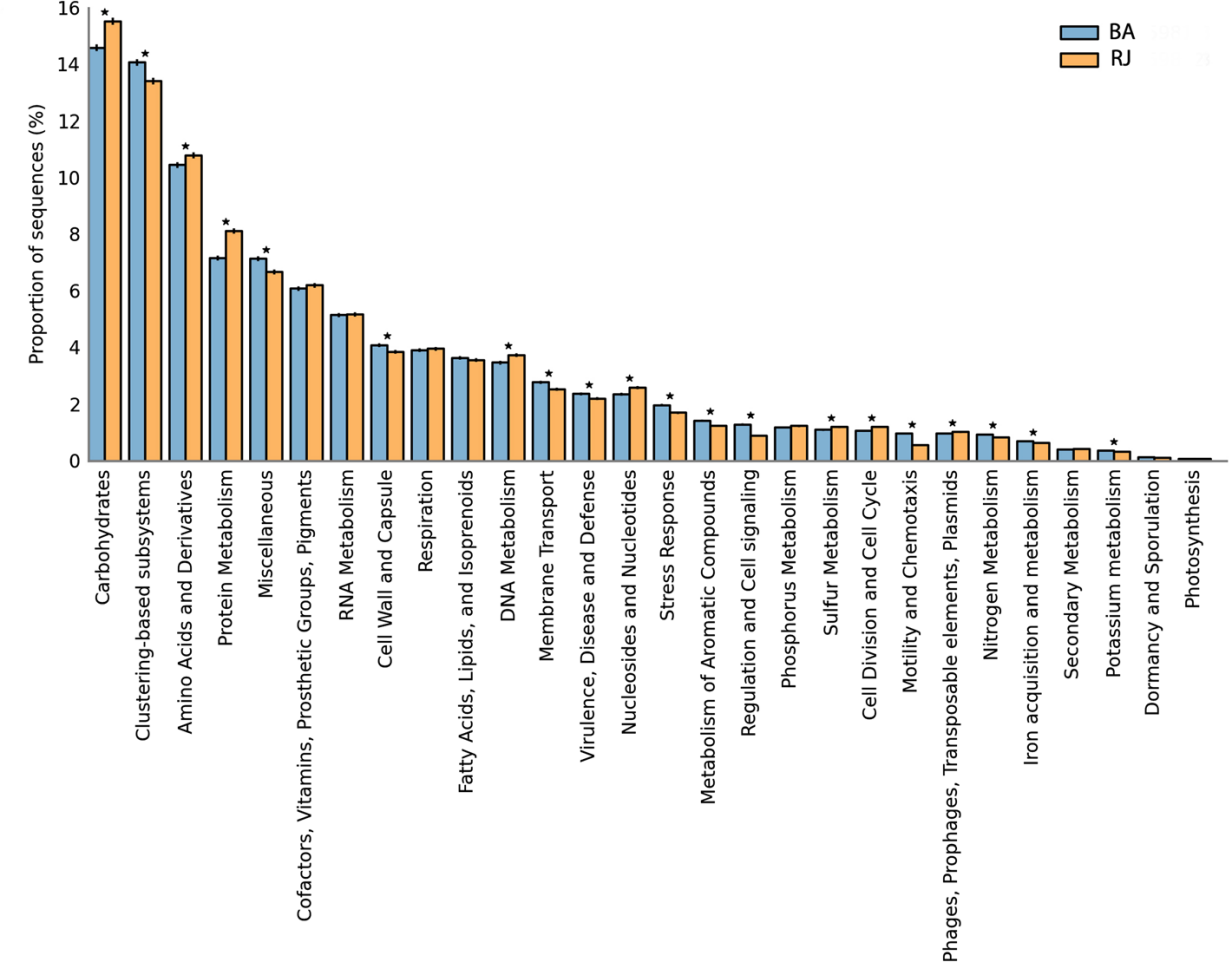
**Profile bar plot showing the relative proportion of RJ (yellow) and BA (blue) sequences classified according the SEED subsystem obtained using the MG-RAST functional profile through STAMP software.** The analysis was conducted considering the entire sample as the parental level and level 1 as the profile level. (*) *p*-value ≤ 0.01.

Subsystem-based annotations (SEED) were performed to analyze the metabolic and physiological conditions in the metagenome of the mangrove samples (Figure 3). A wide variety of environmental gene tags (EGTs) from several metabolic routes were found. The mangroves were found to have a similar profile in terms of the percentages of genes from all of the SEED terms identified. Moreover, the MG-RAST (Figure 3) and MEGAN (Additional file 3: Figure S3) analyses gave similar patterns for both groups of mangrove samples.

The RJ sample was found to have a significantly higher number of EGTs related to carbohydrate metabolism (Figure 3). Genes commonly found in other metagenomes related to amino acid metabolism, cellular respiration, and DNA and protein metabolism were also identified. Furthermore, sequences related to virulence, the stress response, membrane transport, cell wall and capsule synthesis, and the metabolism of aromatic compounds were also significantly more abundant in the BA mangrove. A greater number of EGTs related to nitrogen metabolism, potassium metabolism, and iron acquisition and metabolism were also identified in the BA sample. In contrast, genes involved in sulfur metabolism (Figure 3) and CO_2_ fixation (Additional file 4: Figure S4) were found to be more highly represented in the RJ mangrove.

**Figure 4:**
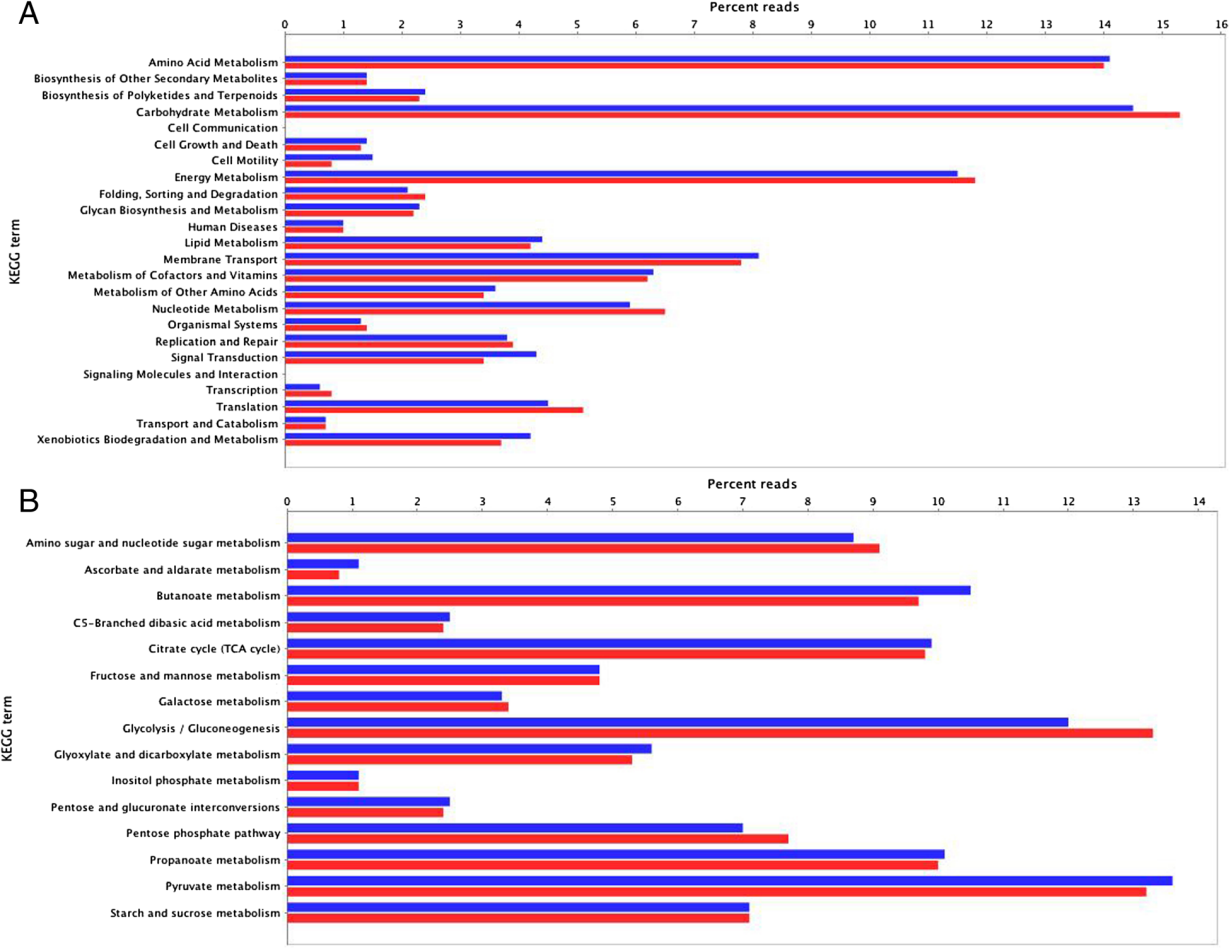
**Functional analysis based on the KEGG main categories. (A)** Relative number of genes from mangrove microbiomes classified according to the KEGG main categories. **(B)** Relative gene diversity within the KEGG pathway for carbohydrate metabolism.

Further analyses of the functional composition of mangrove metagenomes using similarity to a non-redundant protein database against the KEGG metabolic pathways produced similar results (Figure 4A). A deep examination of the carbohydrate metabolic pathways (Figure 4B) revealed such subgroups as fructose and mannose, starch and sucrose, and galactose metabolism with a significant number of putative genes potentially involved in the decomplexation of vegetable biomass.

**Figure 5:**
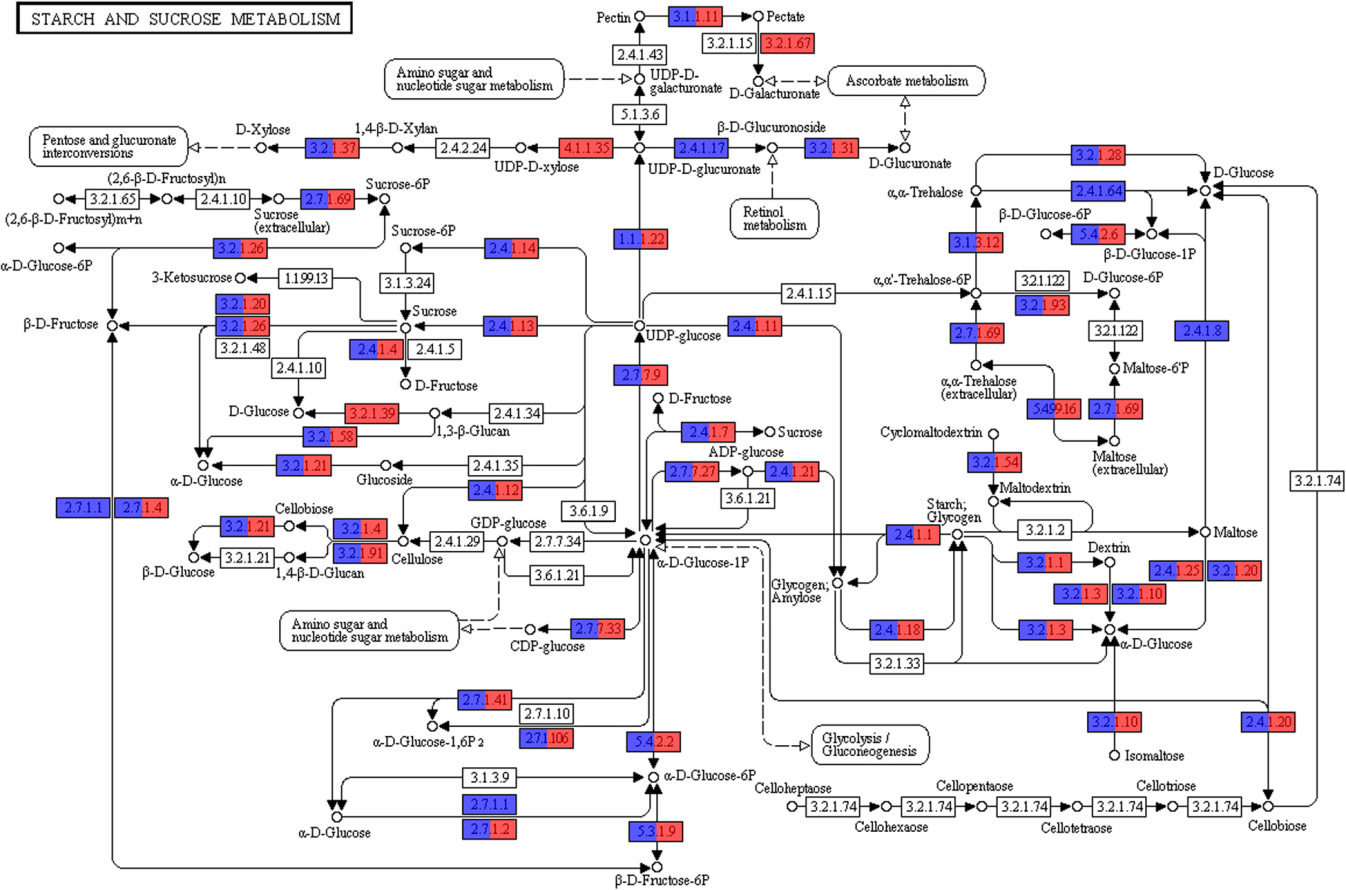
**KEGG sub-pathway for starch and sucrose metabolism.** The color scale represents the number of genes (in logarithmic scale) found for each KEGG entry. RJ (red) and BA (blue).

Several genes related to cellulose degradation were found, including endo-1,4-β-D-glucanases (entry 3.2.1.4; BA: 72 and RJ: 90), 1,4-β-cellobiosidases (3.2.1.91; BA: 4 and RJ: 3), and β-glucosidases (3.2.1.21; BA: 279 and RJ: 228). Sequences classified as putative xylan-degrading enzymes (3.2.1.37, 1,4-β-xylosidases; BA: 23 and RJ: 9) were also identified (Figure 5).

**Figure 6:**
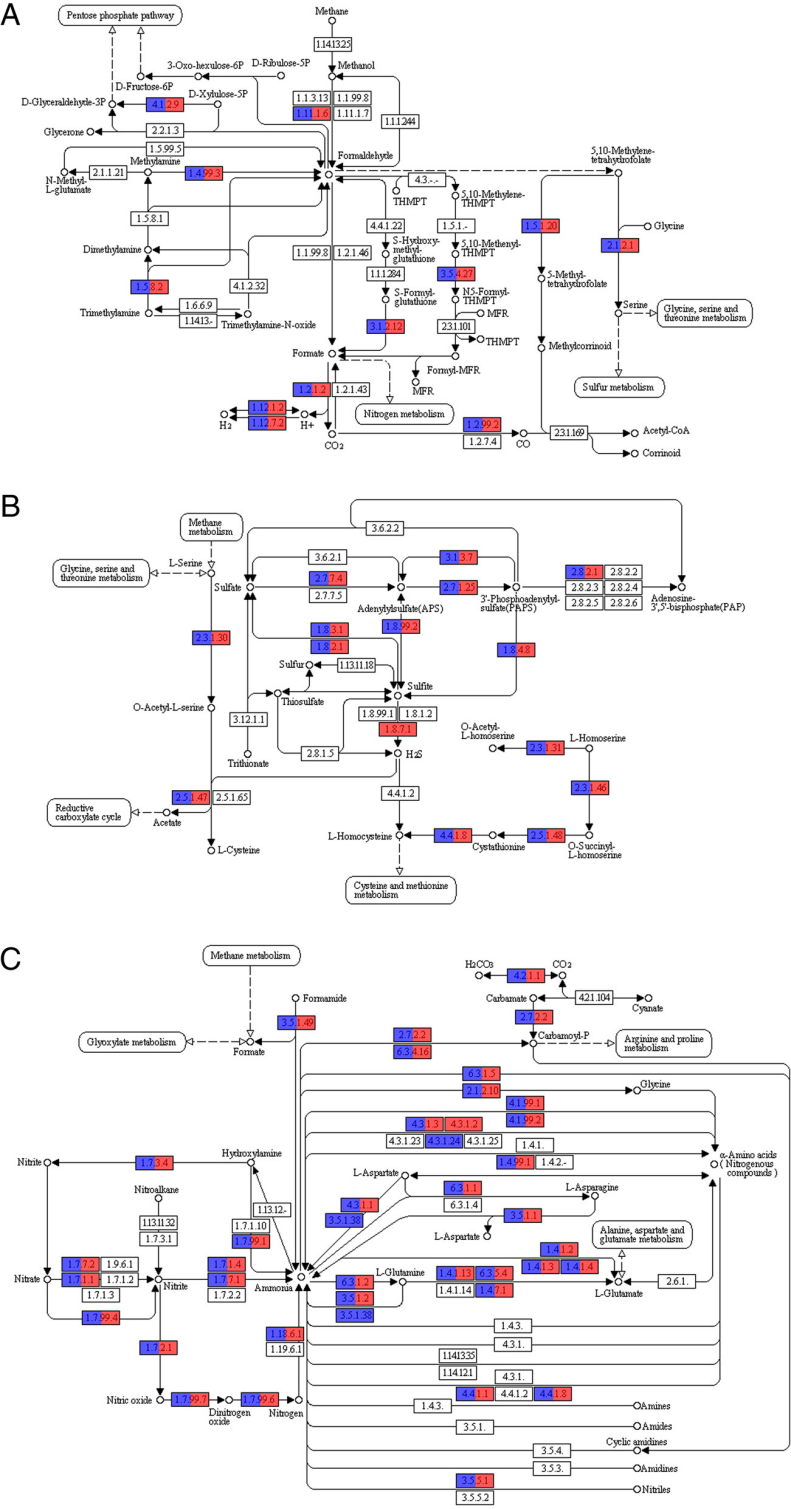
**Functional analysis of the mangrove metagenomes based on the KEGG pathways. (A)** methane metabolism; **(B)** sulfur metabolism, and **(C)** nitrogen metabolism.

The enzymes necessary for the first step of methane metabolism, i.e., the transformation of methane into methanol, were not found. However, several genes related to the conversion of carbon dioxide into carbon monoxide and acetyl-CoA were recovered (Figure 6A). A total of 504 and 562 genes involved in the transformation of formate to CO_2_ were identified in the BA and RJ samples, respectively. There were 75 (BA) and 103 (RJ) hydrogen dehydrogenases responsible for the interconversion of H^+^ and H_2_ identified in the samples.

In terms of sulfur metabolism, several enzymes in the mangroves (BA: 68 and RJ: 72) convert adenylylsulfate into sulfite (Figure 6B). The majority of enzymes involved in nitrogen metabolism are represented between the two mangroves, with the exception of methylaspartate ammonia-lyase (EC number: 4.3.1.2). This enzyme produces ammonia as a subproduct and is present only in the RJ sample (Figure 6C). The pathways leading to ammonia production are well represented by histidine ammonia-lyase (EC: 4.3.1.3), L-tryptophan indole-lyase (EC: 4.1.99.1), D-amino-acid dehydrogenase (EC: 1.4.99.1), and L-cystathionine L-homocysteine-lyase (EC: 4.4.1.8). These enzymes were found at higher abundance levels in the BA sample.

The BA and RJ samples were found to have 60 and 49 nitrogenases involved in biological nitrogen fixation, respectively. *Cyanobacteria*, which were highly abundant in both mangroves, as well as *Chlorobi* (green sulfur bacteria), *Azotobacteraceae* (*Gammaproteobacteria*), *Frankia* (*Actinobacteria*), and *Rhizobia* (*Alphaproteobacteria*) are the diazotrophs involved in this process. Enzymes related to the nitrification were thus identified. However, the denitrification process, which reduces nitrates to nitrogen gas, has an even higher number of sequences in the two mangroves. The bacteria involved in this process are anaerobic and use nitrates as an oxygen alternative to the final electron acceptor in the respiration cycle.

Metabolic pathway comparative analysis with the KEGG database indicated the presence of 202 and 183 alcohol dehydrogenases (EC number: 1.1.1.1) in BA and RJ samples, respectively, which are involved in polycyclic aromatic degradation. Additionally, 27 and 6 salicylate 1-monooxygenases from the same pathway (EC number: 1.14.13.1) were found in the BA and RJ samples, respectively. These enzymes play a role in 1- and 2- methylnaphthalene degradation.

Naphthalene is considered a PAH and is known to cause human disease (IARC, 2002). After evaluating the metabolism of xenobiotics by the cytochrome P450 pathway, two important enzymes for naphthalene metabolism were identified: glutathione dehydrogenase, which had 117 and 40 hits for BA and RJ, respectively, and benzo[*a*]pyrene-4,5-oxide hydratase, which had 14 and 10 hits for BA and RJ, respectively.

### Carbohydrate-active enzyme analysis

Using the CAZy database (http://www.cazy.org), the two mangrove samples were found to have the same metabolic potential to hydrolyze carbohydrates, including cellulosic compounds (cellulose, hemicellulose, pectin, xylan, among others) (Additional file 5: Table S1). This analysis identified more than 2,900 Environmental Gene Tags (EGTs) from 110 different CAZy families, most of which were from *Proteobacteria*. Some CAZy families were only found in one mangrove: GH 6, 45, 70, 71, 72, and CBM 4 were only found in the RJ sample, while GH 11, 46, 79, 81, CE 5, and CBMs 15, 25, and 33 were only found in the BA sample.

A wide diversity of GH (glycosyl hydrolase) families was also identified: 54 and 56 different GH families were found in the BA and RJ mangroves, respectively. GH families with cellulolytic enzymes involved in the degradation of plant cell walls were found in both metagenomes, and only a few members of GH 6 and 45 were detected exclusively in the RJ sample. In addition, enzymes involved in the hydrolysis of hemicellulose/pectin and xylan side chains, including the β-galactosidases (GH 2, 27, 35), the xylanases (GH 10, 26, 43), the acetylxylan esterases (CE 4), the α-mannosidases (GH 38), the α-xylosidases (GH 31), the pectin methylesterases (CE 8), the α-L-rhamnosidases (GH 78), the α-glucuronidases (GH 67), and the pectin lyases (PL 1), were identified (Additional file 5: Table S1).

The RJ and BA mangroves also possess a wide diversity of carbohydrate-binding modules (CBM), which may promote the interaction between a given enzyme and its target substrate, thereby increasing its catalytic efficiency. Important modules for the degradation of cellulose were found (CBM4/9/16/22). The CBM module with the most identified members was CBM50, known as the LysM domain.

In total, 58 families of glycosyl hydrolases, 23 families of glycosyltransferases, 5 families of carbohydrate esterases, and 3 families of polysaccharide lyases were identified in both mangrove microbiomes.

When comparing mangroves to other host-associated and environmental metagenomes (Additional file 5: Table S1), including those from termites, pandas (Zhu et al. 2011), marine water systems (DeLong et al. 2006) and soil, some GHs involved in the hydrolysis of cellulosic/hemicellulosic compounds were found exclusively in the mangroves (GH 6, 12, 17, 44, 46, 47, 62, and 76). Several CBMs important in the recognition of cellulosic compounds were exclusive to the mangroves (CBM 2, 3 and 15). Families GH 3 and GH 13, which contain a large range of glycosidases able to hydrolyze complex carbohydrates into oligosaccharides, were abundant.

Crucial carbohydrate degradation-related domains, including the dockerin and cohesin domains, were found in both mangroves; the cohesins were highly represented (Additional file 5: Table S1). In other metagenomes, only one dockerin (in the marine metagenome) and few cohesins (in the panda and marine metagenomes) were detected. Even in metagenomes specialized in cellulose degradation, such as the termite metagenome, no dockerin/cohesion sequences were identified, further showing the remarkable potential of the mangrove environment for enzyme identification.

Deep analyses of these results also identifies microorganism sequences specialized for cellulose/biomass degradation. Sequences belonging to *Anaerolinea thermophila*, which is a filamentous and thermophilic bacterium, were the most abundant in both mangrove samples (30 CAZy sequences) (Additional file 6: Table S2). Interestingly, these results also showed the identification of CAZy sequences from *Fibrobacter succinogenes* in both microbiomes (RJ: CBM 4, 9, 16, and 22; and BA: GH 30). Sequences from other specialized microorganisms were also found, including *Sorangium cellulosum*, *Solibacter usitatus* and *Spirochaeta thermophila.*

### Comparative metagenomic analysis

**Figure 7:**
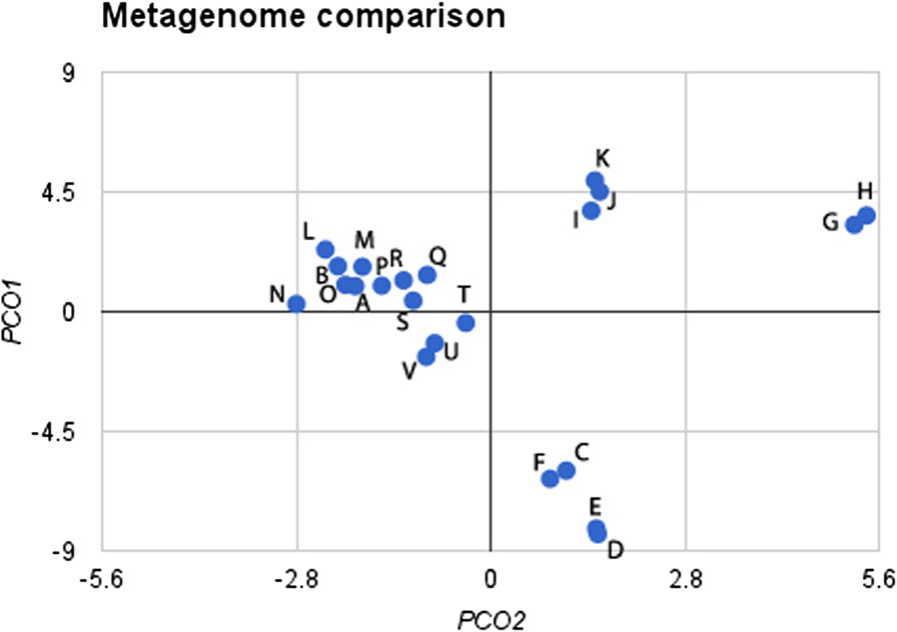
**Comparative analysis of BA and RJ mangroves, water, soil, and host-associated metagenomes obtained through principal component analysis based on SEED subsystems determined using MG-RAST.** The letters indicate the following metagenomes, with the respective MG-RAST acession number: **(A)** BA mangrove (4485982.3), **(B)** RJ mangrove (4485981.3), **(C)** Pacific Ocean, upper mesopelagic 500 m (4441057.3), **(D)** Pacific Ocean, upper euphotic 70 m (4441057.4), **(E)** Pacific Ocean, upper euphotic (4442500.3), **(F)** Pacific Ocean, oxygen minimum layer (4442500.4), **(G)** Lean mouse cecum (4440463.3), **(H)** Obese mouse cecum (4440464.3), **(I)** Human feces (4440939.3), **(J)** Human feces (4440941.3), **(K)** Termite gut (4442701.3), **(L)** Rice rhizosphere (4449956.3), **(M)** Caribbean sea - open Ocean (4441587.3), **(N)** Tropical forest soil (4446153.3), **(O)** Atlantic Ocean 4,200 m (4441572.3), **(P)** Agricultural soil (4441091.3), **(Q)** Water mangrove (4441598.3), **(R)** Atlantic Ocean surface (4441584.3), **(S)** Pacific Ocean 2,736 m (4441594.3), **(T)** Acid mine drainage biofilm (4441138.3), **(U)** Pacific Ocean coast (4443713.3), **(V)** Ocean coast (4443702.3).

The functional information provided by SEED was used to compare the Brazilian mangrove metagenome to the published metagenomes of other environments, including soil, water, and host-associated environments. Principal component analysis with only the first two components could explain 77.2% of the data variance. The resulting plot (Figure 7) showed that the BA and RJ mangroves formed a cluster near the metagenomes of water and soil. The metagenomes from the Caribbean Sea (open ocean), the Atlantic Ocean (4,200 m), agricultural soil, tropical forest soil, and rice rhizospheres were the most similar to the mangrove metagenome. The host-associated metagenomes formed two main groups: one formed by the human and termite sequences, and the other formed by the mouse sequences. Additionally, the metagenomes of euphotic, mesopelagic and ocean regions with a minimum oxygen layer were similar to these functional categories and formed a cluster that was separate from the others.

## Discussion

Mangrove ecosystems are coastal wetlands that are dominated by woody plants across a gradient of latitudes (30 °N to 37 °S), tidal heights (>1 m to <4 m), geomorphologies (oceanic islands to riverine systems), sedimentary environments (peat to alluvial), climates (warm temperate to both arid and wet tropics), and nutrient availabilities (oligotrophic to eutrophic) (Feller et al. 2010). These diverse ecosystems are critical not only for sustaining biodiversity but also for their direct and indirect benefits for human activity (Feller et al. 2010; Koch et al. 2009; Walters et al. 2008). Brazil has 7,408 km of coastline, 6,786 km of which contain mangrove forests. Brazil’s mangroves cover approximately 25,000 km^2^ (Schaeffer-Novelli et al. 200).

Unfortunately, the diversity of global mangroves has been continually declining over the past four decades (Butchart et al. 2010). The world’s total mangrove area has decreased by approximately 35% in the past 30 years (Giri et al. 2011). The current estimation of mangrove forest areas in the world is less than half of what it once was (Spiers et al. 1999; Spalding et al. 1997) and most of the remaining areas are already in a degraded state (Giri et al. 2011). Predictions suggest that a full 100% of the mangrove forests, along with their vast microbial diversity and intrinsic benefits, may be lost within the next 100 years, given the accelerated loss due to both human encroachment and environmental changes (Duke et al. 2007).

Our results are consistent with these predictions; the impacted BA mangrove was less diverse than the well-preserved RJ mangrove. The estimated disappearance rates for mangrove areas vary from between 1 and 8% per year, which is as high as or even higher than the disappearance rates for tropical wet forests. These rates will likely continue to increase, unless mangrove forests can become protected as a valuable resource (Alongi et al. 2002). The exploration of the biotechnological potential of mangroves worldwide, as a source for the discovery of new or improved enzymes and microorganisms for second-generation biofuel production, would have several simultaneous benefits. It could result in the increased protection and preservation of these ecosystems, leading to the maintenance of their ecological and biotechnological potential. It will therefore be useful to optimize and increase the production and consumption of biofuels based on agricultural residues and to thereby decrease the consumption of fossil fuels.

Given their role in vegetable biomass degradation, the neglected mangrove ecosystems have significant biotechnological potential and represent an excellent microbiome for the study of cellulolytic microorganisms and biomass degrading enzymes. The unique characteristics of mangroves include their differences in salinity, oxygen content and nutrients, which are responsible for the high level of diversity and exclusivity of the microorganisms (Gao et al. 2010).

A main goal of modern biotechnological approaches is to access the microbes from unusual or specialized ecological niches, such as mangrove habitats, the rumens of herbivorous animals (Hess et al. 2011; Zhu et al. 2011; Brulc et al. 2009) and the guts of arthropods (Warnecke et al. 2007). All of these microbiomes have the metabolic potential to hydrolyze cellulolytic compounds. A clear difference between searching for enzymes and microorganisms in the mangroves versus in host-associated environments is that the latter represent “closed” environments, with minimal fluctuations in pH, temperature and other environmental parameters that might thus produce a more limited level microbial diversity. The greater microbial diversity of the mangroves and their associated microbial communities may be due to the numerous daily stresses that these organisms face (Xu 2011; Pointing et al. 1999).

Certain microorganisms have developed unique metabolic pathways, ecological adaptability, defenses, ability to communicate, and degradation and predation functions. These microorganisms might therefore produce and secrete effective molecules or enzymes with promising biotechnological applications (Xu 2011). Mangroves sequester up to 25.5 million tonnes of carbon per year (Ong 1993) and provide more than 10% of the essential organic carbon to the global oceans (Dittmar et al. 2006) as such, they are considered an environment of intense carbon flux. Additionally, the wealth and diversity of the microbial community associated with vegetal decomposition (Sahoo and Dhal 2009) makes this unique ecological habitat a potential source of microorganisms and enzymes involved in cellulose decomplexation and sugar release.

Other groups have described the results of mangrove sequencing (Gomes et al. 2008; Andreote et al. 2012; Santos et al. 2011; Dias et al. 2010) and have used isolation as a strategy to assess the biodiversity and enzymatic potential of mangrove microorganisms for their use in biotechnological purposes. In this work, microorganism cultivation and a metagenomic approach were used to explore the biodiversity and biotechnological potential of the mangroves.

According to Gao et al. (2010), microorganisms from extreme environments and their unique enzymatic repertoire have a great potential value for use in biotechnology. The mangrove ecosystem is not generally considered an extreme environment, but its unique environmental features make it a similarly attractive system to look for microorganisms with useful applications. In a recent review, Xu (2011) describes several natural products that can be extracted from mangrove-associated microorganisms, with a particular focus on their bioactivities. Although the biotechnological potential of mangrove microorganisms has been an underrepresented research topic, two cellulase genes were identified in the bacterial isolates of the mangrove soils and were subsequently characterized (Gao et al. 2010; Yang et al. 2001), demonstrating the potential of this environment to degrade cellulosic compounds.

In our study, 179 (60.5%) and 117 (39.5%) microorganisms (bacteria, yeast and filamentous fungi) were isolated from the RJ and BA samples, respectively (Figure 1A), including 21 filamentous fungi with cellulolytic potential. Sahoo and Dhal (2009) reviewed the microbial diversity of mangrove ecosystems and highlighted the crucial role of these fungi as the primary microorganisms responsible for the decomposition of cellulosic material. These organisms can tolerate the high levels of phenolic compounds found on mangrove leaves that commonly inhibit the growth of other microorganisms (Raghukumar et al. 1995). They are therefore able to begin the process of vegetative material decomposition and permit the secondary colonization of bacteria and yeasts, which can then further decompose the organic matter (Sahoo and Dhal 2009). However, little is known about the physiology and biochemistry of mangrove fungi. This lack of knowledge could limit the isolation and study of the role of such microorganisms in nutrient recycling (Sahoo and Dhal 2009).

When considering the microbial diversity as a whole, the alpha diversity indicated that the RJ mangrove (alpha-diversity = 974.385 species) is more diverse than the BA mangrove (alpha-diversity = 847.721 species). This index could explain the higher number of microbial isolates in the RJ mangrove compared to the BA mangrove (Figure 1A). This result is also consistent with the conservation states of RJ and BA (RJ is highly preserved, whereas BA has a low to medium level of degradation) because the degradation of mangrove areas is closely related a decrease in their biodiversity levels (Giri et al. 2011).

Considering the different taxonomic groups found on the mangroves using metagenomic approaches, the high level of diversity found in the mangrove samples is expected because multiple species may perform both different and similar roles (Hooper er al. 2005). They can thus provide stability to the mangrove ecosystem during times of environmental disturbance (Gomes et al. 2008). The identified predominance of *Gammaproteobacteria* is consistent with previous data from Andreote et al. (2012) and Santos et al. (2011), indicating the dominance of this group in both natural and impacted environments. Dias et al. (2010) reported that both dominant and low-density communities are influenced by environmental changes, indicating that these groups are essential in the maintenance of ecosystem functionality during daily or yearly changes in climate.

Sequences belonging to *Deltaproteobacteria*, which have not been commonly found in seawater and soil samples, were identified in the preserved RJ mangrove ecosystem. It may be that the anaerobic conditions of the RJ mangrove provide a selective pressure leading to the prevalence of microbial groups such as sulfate-reducing bacteria (Taketani et al. 2010a; Taketani et al. 2010b).

In relation to the general results related to the functional aspects of the mangroves, the RJ sample presented more genes involved in sulfur metabolism (Figure 3) and CO_2_ fixation (Additional file 4: Figure S4). The biogeochemical cycles acting on the BA and RJ mangroves were evaluated, particularly in regards to their methane, sulfur and nitrogen metabolism. Although these environments are predominantly anoxic, sea flooding produces occasional aerobic conditions, thereby providing an opportunity for the nitrification process to occur. Enzymes involved in the direct conversion of sulfite into H_2_S through sulfite reductase were only identified in the RJ mangrove. It is important to note that the RJ sample had a higher number of *Deltaproteobacteria*, which are related to sulfate reduction. This finding is evidence for the importance of sulfur metabolism in the RJ mangrove.

A higher number of enzymes involved in the polycyclic aromatic degradation were found in the BA sample. Due to their high primary productivity, abundant detritus, rich organic matter and anoxic/reduced conditions, the mangroves are preferential sites for the uptake and preservation of PAHs, resultant of high anthropogenic impact (Bernard et al. 1996). The polycyclic aromatic compounds have several possible mechanisms for environmental release, including volatilization, photo-oxidation, chemical oxidation, bioaccumulation, adsorption on soil particles, leaching, and microbial degradation. Importantly, microbial degradation is believed to be the most important process for the successful removal of such compounds. The microorganisms of hydrocarbon-contaminated areas have a higher biodegradation potential than those from non-contaminated environments, especially in terms of their acclimation function and their adaptation to contaminated areas (Yun et al. 2008). The atmospheric pollution of the urban area of Porto Seguro may be the reason why we found a higher number of enzymes involved in the degradation of polycyclic aromatic compounds in the BA mangrove. Indeed, these contaminants are spreading along the coast where the BA mangroves are found. Peixoto et al. (2011) have demonstrated that the total level of PAHs is related to a higher abundance of Actinobacteria and Alphaproteobacteria in the samples, which is in agreement with the results for BA mangrove. Additionally, a recent study (Arias et al., 2010) showed that the proximity to sources was the most important determining factor for the distribution of PAHs, with the higher concentrations being greater in samples collected near industrial areas.

Several cellulolytic enzymes were found on the mangroves, some of them being only found in one mangrove. This finding indicates that exploring different mangrove areas and samples simultaneously might provide a higher coverage of the different enzyme families. Given that Brazil contains 7% of the world’s total mangrove area (Giri et al. 2011) spread across various regions with distinct environmental conditions, the potential for discovery is remarkable. The mangrove environment should be considered a good source of carbohydrate-degrading enzymes.

The enormous GH variability found highlights the great potential of this environment for the identification of polysaccharide and cellulosic-degrading enzymes. These enzymes are very important in biomass decomplexation for biofuel production because they open the cellulosic fibers. The abundance of the GH and CBM families identified in the mangroves reflects their high capacity to attach to, to metabolize and to degrade a diverse array of cellulosic substrates. In contrast, only one family likely to bind to cellulose was found in the bovine rumen (Brulc et al. 2009).

The GH families 3 and 13, which were found on the mangroves, were also found to be abundant in previously reported metagenomes (Pope et al. 2010; Brulc et al. 2009; Warnecke et al. 2007). The GH 3 family is known to have a bi-functional mechanism of action and has been reported to have cellobiase activity (Faure et al. 2011).

The specificity of cohesin-dockerin interactions is critically important for the assembly of the multienzyme cellulolytic complex (Slutzki et al. 2012). This so-called ‘cellulosome’ is defined as a multienzyme and is highly active against crystalline cellulose and related plant cell wall polysaccharides (Cha et al. 2007). Each cohesin domain consists of a subunit-binding domain that interacts with a docking domain (dockerin) for each catalytic component of the cellulosomal enzyme (Cha et al. 2007). The efficient enzymatic degradation of insoluble polysaccharides requires both the tight interaction between the enzymes and their substrates and the cooperation of multiple enzymes to enhance the hydrolysis, leading to the formation of a complex structure (Slutzki et al. 2012; Cha et al. 2007; Patthra et al. 2006; Takagi et al. 1993). The attachment of the cellulosome to its substrate is mediated by a cellulose-binding module (CBM) that comprises part of the cellulosome subunit (Fontes et al. 2010; Doi et al. 2004). This structure was widely found in the two mangrove samples (Additional file 5: Table S1). Therefore, all of the necessary molecular machinery for cellulosome production, efficient cellulose degradation, and sugar release were found to be present in the mangrove ecosystem.

The CBM module with the most identified members was CBM50, known as the LysM domain. This domain has multiple functions, including signaling and recognition in host-microbe interactions. It is involved in symbiotic and cell wall degradation (Bensmihen et al. 2011).

Among the species identified in our study is *A. thermophila,* which could be a good candidate for further studies because there are only a few reports about it. Moreover, it is known that this bacterium can produce an increase in the carbohydrate concentrations when growing in the up flow of an anaerobic sludge blanket reactor (Sekiguchi et al. 2003). According to these results, *A. thermophila* has a promising but yet-unknown cellulolytic potential that should be explored in future work. *Fibrobacter succinogenes* found in both microbiomes was prominent in the rumen of herbivores and is considered to be specialized in using only cellulose as its carbon source (Suen et al. 2010). In this case, polysaccharide-degrading strategy is different from that of other cellulolytic microorganisms, as it does not possess a vast repertoire of cellulases or cellulosomal structures. *F. succinogenes* adheres to solid cellulosic substrate, most likely forming a biofilm on the cellulose surface (Suen et al. 2010). Sequences from other specialized microorganisms were also found, including *Sorangium cellulosum*, a cellulolytic myxobacterium that can efficiently degrade many types of polysaccharides such as cellulose (Wang et al. 2007), *Solibacter usitatus*, an *Acidobacteria* found in soils and sediments worldwide, with four times more genes for carbohydrate metabolism and transport than other known *Acidobacteria* (Challacombe et al. 2011) and *Spirochaeta thermophila*, a thermophilic, free-living, and cellulolytic anaerobe, found only in the RJ mangrove (Angelov et al. 2011).

The biodiversity and biotechnological potential of two Brazilian mangroves were assessed through cultivation and metagenomic approaches. The results presented here demonstrate the great biotechnological potential of this unique but endangered environment.

## Competing interests

The authors declare that they have no competing interests.

## Authors’ contributions

Performed the experiments: MB; Analyzed the experimental data: WOB, LS; Analyzed the statistical, taxonomic, and comparative data: CET; Analyzed the functional data: CET, LS, WOBS; Contributed for the reagents/materials: ATRV, JAG, MHV, and WOBS; Wrote the manuscript: CET, LS, and WOBS; Participated in the design and coordination of the study: ATRV, JAG, MHV. All authors read and approved the final manuscript.

## Supplementary Material

Additional file 1: Figure S1 Location of the collected BA and RJ mangrove samples. Click here for file

Additional file 2: Figure S2 Profile bar plot showing the relative proportion of RJ (yellow) and BA (blue) taxa obtained using the MG-RAST taxonomic profile through STAMP software. The analysis was conducted considering the domain as the parental level and phylum as the profile level. *Bacteria* in red. (*) p-value ≤ 0.01. Click here for file

Additional file 3: Figure S3 Profile bar plot showing an overview of the relative proportion of RJ (red) and BA (blue) sequences classified according the SEED subsystem obtained using the MEGAN software. Click here for file

Additional file 4: Figure S4 Profile bar plot showing the relative proportion of RJ (yellow) and BA (blue) taxa obtained using the MG-RAST taxonomic profile through STAMP software. The analysis was conducted considering the entire sample as the parental level and level 2 as the profile level. (*) p-value ≤ 0.01. Click here for file

Additional file 5: Table S1 Comparative analysis of CAZy families belonging to the mangroves and other metagenomic microbiomes. Click here for file

Additional file 6: Table S2 The main taxonomic groups found in the mangroves and their correspondence to each CAZy family. Click here for file
