# Supplementary figures and images for: A potential source for cellulolytic enzyme discovery and environmental aspects revealed through metagenomics of Brazilian mangroves
Source: AMB Express. 2013 Oct 26;3:65. doi: 10.1186/2191-0855-3-65 (PMC3922913; doi:10.1186/2191-0855-3-65)

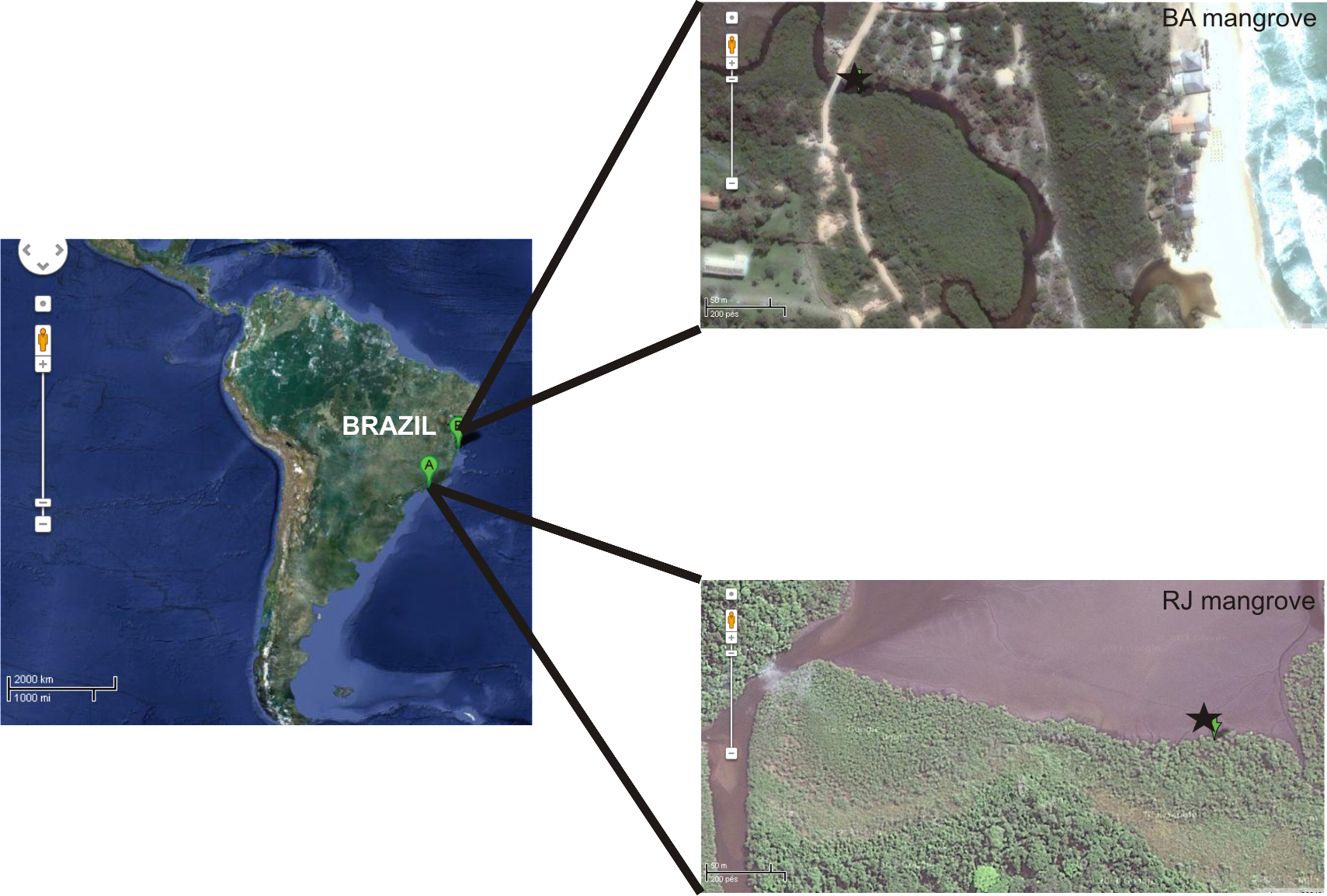

Supplement: Additional file 1: Figure S1 — Location of the collected BA and RJ mangrove samples. [file 2191-0855-3-65-S1.jpeg]

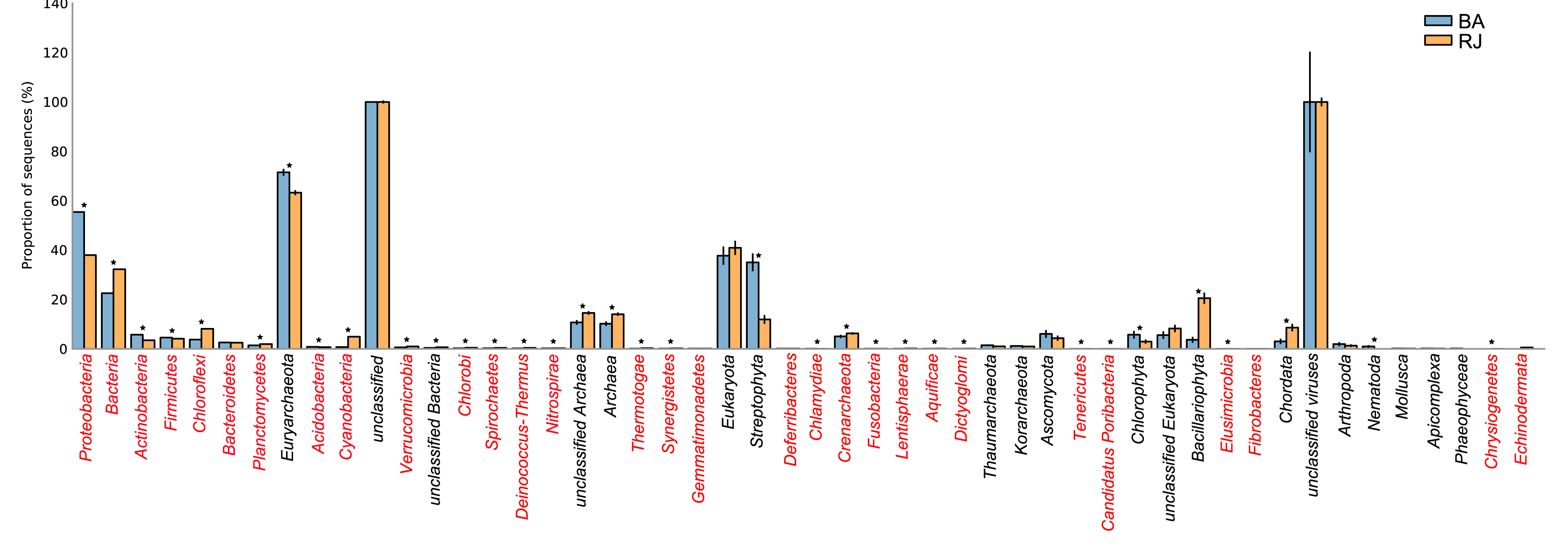

Supplement: Additional file 2: Figure S2 — Profile bar plot showing the relative proportion of RJ (yellow) and BA (blue) taxa obtained using the MG-RAST taxonomic profile through STAMP software. The analysis was conducted considering the domain as the parental level and phylum as the profile level. Bacteria in red. (*) p-value ≤ 0.01. [file 2191-0855-3-65-S2.jpeg]

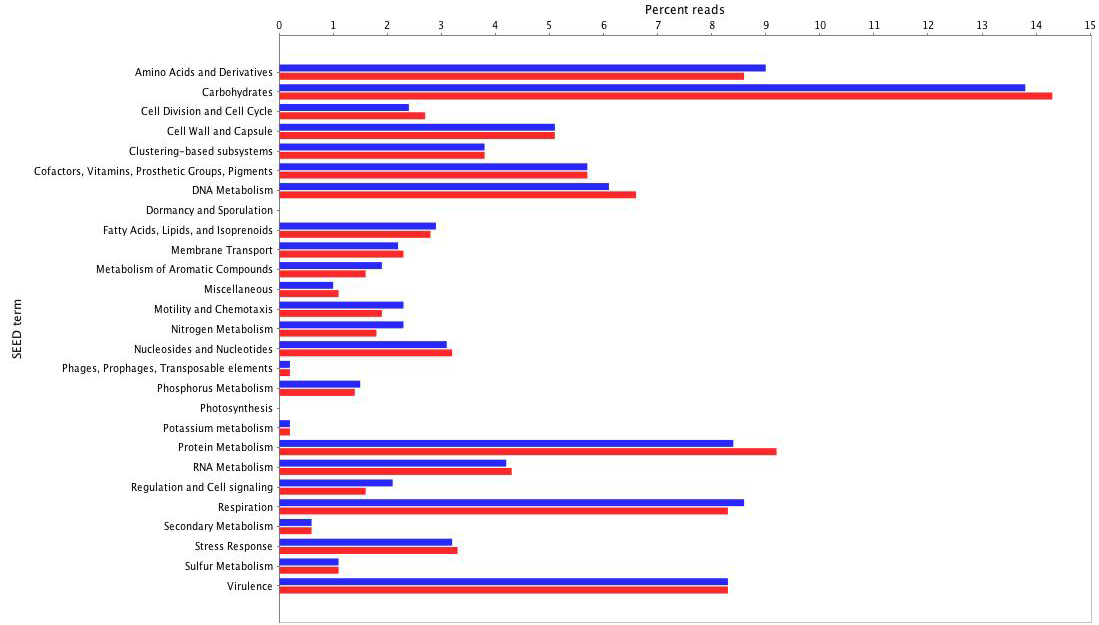

Supplement: Additional file 3: Figure S3 — Profile bar plot showing an overview of the relative proportion of RJ (red) and BA (blue) sequences classified according the SEED subsystem obtained using the MEGAN software. [file 2191-0855-3-65-S3.jpeg]

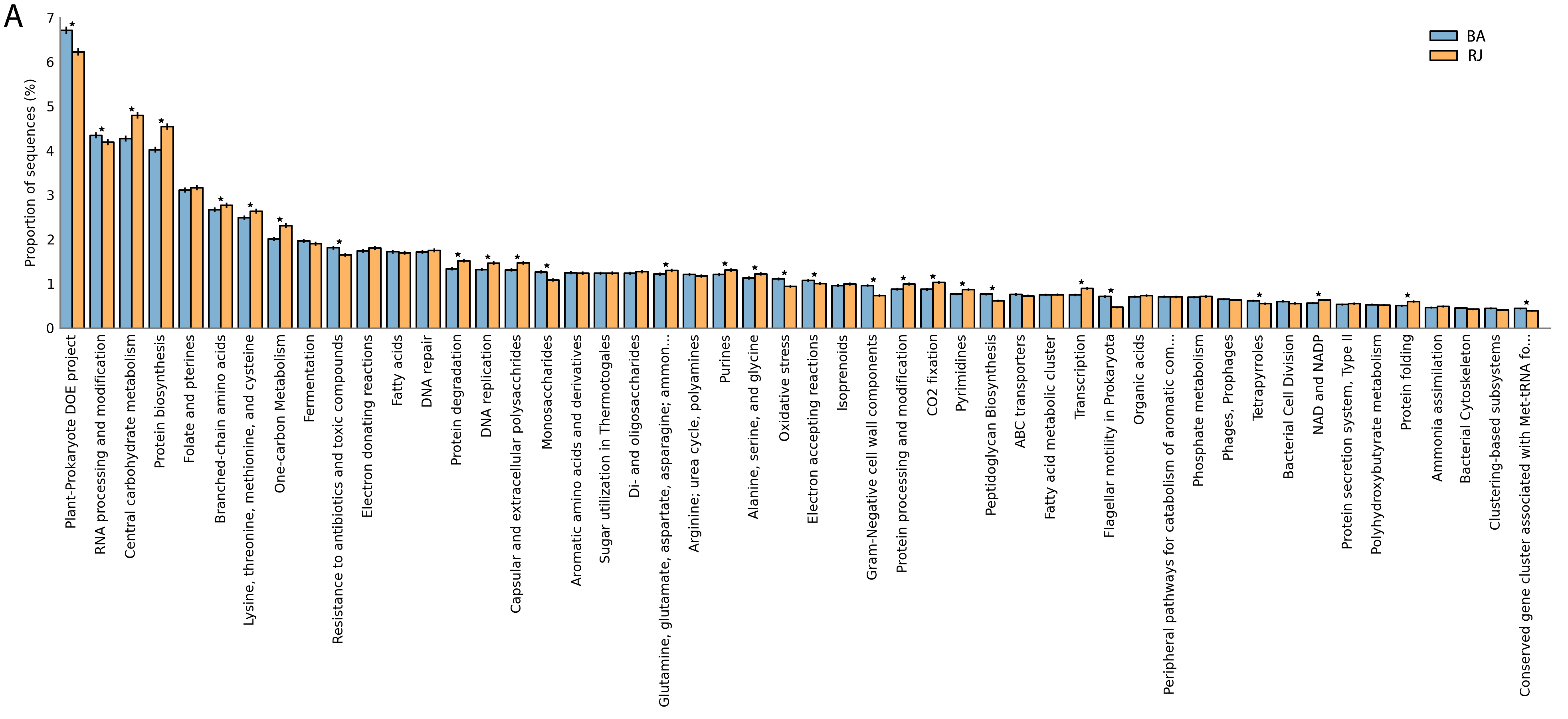

Supplement: Additional file 4: Figure S4 — Profile bar plot showing the relative proportion of RJ (yellow) and BA (blue) taxa obtained using the MG-RAST taxonomic profile through STAMP software. The analysis was conducted considering the entire sample as the parental level and level 2 as the profile level. (*) p-value ≤ 0.01. [file 2191-0855-3-65-S4.jpeg]
