# Supplementary material for: A potential source for cellulolytic enzyme discovery and environmental aspects revealed through metagenomics of Brazilian mangroves
Source: AMB Express. 2013 Oct 26;3:65. doi: 10.1186/2191-0855-3-65 (PMC3922913; doi:10.1186/2191-0855-3-65)
Supplement: Additional file 5: Table S1 — Comparative analysis of CAZy families belonging to the mangroves and other metagenomic microbiomes. [file 2191-0855-3-65-S5.docx]

Table S1. Comparative analysis of CAZy families between mangroves and other metagenomic microbiomes.

| **CAZy family** | **MABA^a^** | **MARJ^a^** | **Termite1^a^** | **Termite2^a^** | **Panda1^a^** | **Panda2^a^** | **Panda5^a^** | **SoilL^a^** | **Eupho^a^** | **Meso^a^** | **Omin^a^** |
| --- | --- | --- | --- | --- | --- | --- | --- | --- | --- | --- | --- |
| **Glycoside Hydrolase** |  |  |  |  |  |  |  |  |  |  |  |
| GH1 | 54 | 60 | 27 | 4 | 12 | 11 | 18 | 1 | 2 | 0 | 2 |
| GH2 | 36 | 35 | 32 | 8 | 1 | 3 | 0 | 3 | 0 | 0 | 0 |
| GH3 | 151 | 127 | 109 | 7 | 5 | 2 | 4 | 2 | 4 | 3 | 4 |
| GH4 | 39 | 52 | 19 | 0 | 3 | 2 | 0 | 0 | 1 | 2 | 0 |
| GH5 | 35 | 29 | 125 | 5 | 0 | 0 | 1 | 0 | 1 | 1 | 4 |
| GH6 | 0 | 3 | 0 | 0 | 0 | 0 | 0 | 0 | 0 | 0 | 0 |
| GH8 | 11 | 4 | 21 | 0 | 0 | 0 | 1 | 0 | 1 | 0 | 0 |
| GH9 | 12 | 9 | 43 | 0 | 0 | 0 | 0 | 0 | 0 | 0 | 0 |
| GH10 | 31 | 21 | 102 | 2 | 0 | 1 | 0 | 0 | 0 | 1 | 0 |
| GH11 | 1 | 0 | 19 | 0 | 0 | 0 | 0 | 0 | 0 | 0 | 0 |
| GH12 | 3 | 1 | 0 | 0 | 0 | 0 | 0 | 0 | 0 | 0 | 0 |
| GH13 | 288 | 322 | 121 | 5 | 10 | 17 | 14 | 11 | 3 | 2 | 3 |
| GH15 | 57 | 20 | 0 | 0 | 0 | 0 | 0 | 1 | 1 | 0 | 0 |
| GH16 | 10 | 30 | 6 | 0 | 0 | 2 | 4 | 0 | 0 | 3 | 2 |
| GH17 | 5 | 6 | 0 | 0 | 0 | 0 | 0 | 0 | 0 | 0 | 0 |
| GH18 | 12 | 6 | 28 | 0 | 0 | 0 | 0 | 0 | 0 | 1 | 0 |
| GH19 | 3 | 1 | 1 | 0 | 0 | 0 | 0 | 1 | 1 | 0 | 0 |
| GH20 | 35 | 28 | 18 | 4 | 1 | 1 | 2 | 0 | 0 | 0 | 1 |
| GH24 | 1 | 8 | 0 | 0 | 0 | 2 | 2 | 3 | 3 | 0 | 0 |
| GH25 | 9 | 11 | 4 | 0 | 0 | 2 | 4 | 1 | 0 | 1 | 0 |
| GH26 | 9 | 10 | 20 | 2 | 0 | 0 | 0 | 0 | 0 | 0 | 0 |
| GH27 | 40 | 9 | 13 | 2 | 0 | 4 | 7 | 0 | 0 | 0 | 0 |
| GH28 | 17 | 17 | 15 | 0 | 0 | 0 | 0 | 0 | 0 | 0 | 0 |
| GH29 | 71 | 77 | 12 | 1 | 0 | 0 | 0 | 2 | 1 | 0 | 2 |
| GH30 | 6 | 19 | 13 | 0 | 0 | 0 | 0 | 0 | 0 | 0 | 0 |
| GH31 | 60 | 80 | 36 | 4 | 1 | 0 | 3 | 7 | 1 | 1 | 0 |
| GH32 | 21 | 10 | 2 | 1 | 0 | 5 | 3 | 0 | 1 | 0 | 1 |
| GH33 | 53 | 86 | 0 | 0 | 0 | 0 | 0 | 0 | 0 | 0 | 0 |
| GH35 | 6 | 8 | 7 | 1 | 0 | 0 | 1 | 0 | 0 | 0 | 0 |
| GH37 | 11 | 8 | 16 | 1 | 0 | 0 | 0 | 0 | 1 | 0 | 1 |
| GH38 | 41 | 54 | 18 | 1 | 3 | 3 | 2 | 2 | 0 | 1 | 1 |
| GH39 | 8 | 5 | 13 | 0 | 1 | 3 | 4 | 0 | 0 | 0 | 0 |
| GH42 | 47 | 50 | 33 | 0 | 0 | 6 | 1 | 0 | 0 | 0 | 1 |
| GH43 | 49 | 34 | 63 | 5 | 2 | 6 | 5 | 0 | 0 | 0 | 1 |
| GH44 | 2 | 1 | 0 | 0 | 0 | 0 | 0 | 0 | 0 | 0 | 0 |
| GH45 | 0 | 1 | 6 | 0 | 0 | 0 | 0 | 0 | 0 | 0 | 0 |
| GH46 | 1 | 0 | 0 | 0 | 0 | 0 | 0 | 0 | 0 | 0 | 0 |
| GH47 | 2 | 1 | 0 | 0 | 0 | 0 | 0 | 0 | 0 | 0 | 0 |
| GH51 | 18 | 18 | 13 | 3 | 0 | 1 | 1 | 0 | 0 | 0 | 0 |
| GH52 | 0 | 0 | 3 | 0 | 0 | 0 | 0 | 0 | 0 | 0 | 0 |
| GH53 | 9 | 11 | 20 | 0 | 1 | 3 | 0 | 0 | 0 | 0 | 0 |
| GH56 | 0 | 0 | 0 | 0 | 0 | 0 | 0 | 0 | 1 | 0 | 0 |
| GH57 | 59 | 92 | 52 | 4 | 0 | 0 | 0 | 4 | 1 | 2 | 1 |
| GH62 | 1 | 2 | 0 | 0 | 0 | 0 | 0 | 0 | 0 | 0 | 0 |
| GH63 | 9 | 14 | 0 | 0 | 0 | 0 | 0 | 0 | 0 | 0 | 0 |
| GH65 | 55 | 36 | 11 | 0 | 0 | 0 | 2 | 0 | 0 | 0 | 0 |
| GH67 | 4 | 4 | 6 | 0 | 0 | 1 | 1 | 0 | 0 | 0 | 0 |
| GH70 | 0 | 1 | 0 | 0 | 0 | 0 | 2 | 0 | 0 | 0 | 0 |
| GH71 | 0 | 1 | 0 | 0 | 0 | 0 | 0 | 0 | 0 | 0 | 0 |
| GH72 | 0 | 1 | 0 | 0 | 0 | 0 | 0 | 0 | 0 | 0 | 0 |
| GH73 | 16 | 16 | 7 | 1 | 2 | 3 | 11 | 0 | 2 | 0 | 0 |
| GH76 | 2 | 3 | 0 | 0 | 0 | 0 | 0 | 0 | 0 | 0 | 0 |
| GH77 | 70 | 92 | 34 | 7 | 0 | 2 | 2 | 4 | 0 | 1 | 1 |
| GH78 | 75 | 46 | 7 | 3 | 1 | 0 | 0 | 0 | 0 | 0 | 0 |
| GH79 | 1 | 0 | 1 | 0 | 0 | 0 | 0 | 0 | 0 | 0 | 0 |
| GH81 | 1 | 0 | 0 | 0 | 0 | 0 | 0 | 0 | 1 | 0 | 0 |
| GH85 | 0 | 0 | 0 | 0 | 0 | 0 | 1 | 0 | 0 | 0 | 0 |
| GH88 | 20 | 21 | 19 | 5 | 0 | 2 | 2 | 0 | 0 | 1 | 0 |
| GH89 | 3 | 1 | 2 | 0 | 0 | 0 | 0 | 0 | 0 | 0 | 0 |
| GH102 | 11 | 8 | 0 | 1 | 0 | 0 | 0 | 1 | 0 | 3 | 1 |
| GH104 | 1 | 8 | 0 | 0 | 0 | 2 | 2 | 3 | 3 | 0 | 0 |
|  |  |  |  |  |  |  |  |  |  |  |  |
| **Polysaccharide Lyase** |  |  |  |  |  |  |  |  |  |  |  |
| PL1 | 7 | 2 | 9 | 0 | 0 | 0 | 1 | 0 | 0 | 0 | 0 |
| PL5 | 5 | 4 | 0 | 0 | 0 | 0 | 0 | 0 | 0 | 0 | 0 |
| PL8 | 2 | 1 | 0 | 0 | 0 | 0 | 0 | 0 | 0 | 0 | 0 |
|  |  |  |  |  |  |  |  |  |  |  |  |
| **Glycosyl Transferase** |  |  |  |  |  |  |  |  |  |  |  |
| GT1 | 10 | 7 | 1 | 1 | 0 | 0 | 0 | 0 | 0 | 0 | 0 |
| GT5 | 7 | 23 | 0 | 0 | 0 | 0 | 0 | 5 | 1 | 0 | 0 |
| GT7 | 3 | 2 | 0 | 0 | 0 | 0 | 0 | 0 | 0 | 0 | 0 |
| GT8 | 6 | 4 | 6 | 10 | 0 | 1 | 2 | 2 | 0 | 0 | 0 |
| GT9 | 97 | 109 | 4 | 9 | 0 | 3 | 1 | 6 | 3 | 2 | 8 |
| GT10 | 3 | 3 | 1 | 0 | 0 | 0 | 0 | 2 | 0 | 0 | 0 |
| GT11 | 1 | 1 | 0 | 1 | 0 | 0 | 0 | 0 | 3 | 0 | 0 |
| GT17 | 0 | 1 | 0 | 0 | 0 | 0 | 0 | 0 | 0 | 0 | 0 |
| GT19 | 63 | 45 | 6 | 6 | 0 | 0 | 0 | 1 | 2 | 1 | 1 |
| GT20 | 83 | 73 | 0 | 0 | 0 | 0 | 0 | 0 | 0 | 0 | 1 |
| GT22 | 0 | 3 | 0 | 0 | 0 | 0 | 0 | 0 | 0 | 0 | 0 |
| GT25 | 1 | 1 | 0 | 0 | 0 | 0 | 2 | 0 | 2 | 0 | 0 |
| GT26 | 25 | 27 | 11 | 0 | 3 | 5 | 1 | 8 | 0 | 0 | 0 |
| GT28 | 50 | 61 | 32 | 9 | 2 | 3 | 6 | 2 | 2 | 1 | 3 |
| GT34 | 1 | 0 | 0 | 0 | 0 | 0 | 0 | 0 | 0 | 0 | 0 |
| GT35 | 169 | 202 | 55 | 3 | 4 | 3 | 4 | 1 | 3 | 0 | 0 |
| GT39 | 12 | 20 | 2 | 1 | 2 | 0 | 0 | 6 | 0 | 2 | 3 |
| GT47 | 1 | 1 | 0 | 0 | 0 | 0 | 0 | 1 | 2 | 0 | 0 |
| GT48 | 0 | 2 | 0 | 0 | 0 | 0 | 0 | 0 | 1 | 0 | 0 |
| GT50 | 0 | 1 | 0 | 0 | 0 | 0 | 0 | 0 | 0 | 0 | 0 |
| GT51 | 146 | 101 | 61 | 13 | 1 | 5 | 4 | 4 | 7 | 6 | 2 |
| GT53 | 9 | 1 | 0 | 0 | 0 | 0 | 0 | 0 | 0 | 0 | 0 |
| GT66 | 9 | 12 | 0 | 1 | 0 | 0 | 1 | 0 | 0 | 0 | 0 |
|  |  |  |  |  |  |  |  |  |  |  |  |
| **Carbohydrate Esterase** |  |  |  |  |  |  |  |  |  |  |  |
| CE4 | 126 | 124 | 34 | 6 | 5 | 8 | 11 | 8 | 3 | 4 | 11 |
| CE5 | 2 | 0 | 0 | 0 | 0 | 0 | 0 | 0 | 0 | 0 | 0 |
| CE8 | 12 | 6 | 0 | 0 | 0 | 0 | 0 | 1 | 0 | 0 | 0 |
| CE11 | 57 | 58 | 9 | 6 | 2 | 1 | 1 | 2 | 2 | 4 | 5 |
| CE14 | 86 | 104 | 3 | 5 | 0 | 1 | 1 | 0 | 0 | 4 | 2 |
|  |  |  |  |  |  |  |  |  |  |  |  |
| **Carbohydrate-Binding Module** |  |  |  |  |  |  |  |  |  |  |  |
| CBM2 | 2 | 1 | 0 | 0 | 0 | 0 | 0 | 0 | 0 | 0 | 0 |
| CBM3 | 0 | 1 | 0 | 0 | 0 | 0 | 0 | 0 | 0 | 0 | 0 |
| CBM4 | 7 | 10 | 9 | 0 | 1 | 7 | 9 | 0 | 1 | 0 | 0 |
| CBM5 | 1 | 1 | 0 | 0 | 0 | 0 | 0 | 0 | 1 | 0 | 0 |
| CBM6 | 10 | 8 | 17 | 0 | 0 | 1 | 1 | 0 | 0 | 0 | 2 |
| CBM9 | 7 | 10 | 9 | 0 | 1 | 7 | 9 | 0 | 1 | 0 | 0 |
| CBM12 | 1 | 1 | 0 | 0 | 0 | 0 | 0 | 0 | 1 | 0 | 0 |
| CBM13 | 2 | 2 | 0 | 1 | 0 | 0 | 0 | 0 | 0 | 0 | 0 |
| CBM15 | 1 | 0 | 0 | 0 | 0 | 0 | 0 | 0 | 0 | 0 | 0 |
| CBM16 | 7 | 10 | 9 | 0 | 1 | 7 | 9 | 0 | 1 | 0 | 0 |
| CBM20 | 2 | 2 | 0 | 3 | 0 | 0 | 0 | 1 | 0 | 0 | 0 |
| CBM21 | 1 | 2 | 0 | 0 | 0 | 0 | 0 | 0 | 0 | 0 | 0 |
| CBM22 | 7 | 10 | 9 | 0 | 1 | 7 | 9 | 0 | 1 | 0 | 0 |
| CBM25 | 1 | 0 | 0 | 0 | 0 | 0 | 0 | 0 | 0 | 0 | 1 |
| CBM32 | 31 | 46 | 6 | 3 | 1 | 8 | 5 | 0 | 0 | 0 | 2 |
| CBM33 | 2 | 0 | 0 | 0 | 0 | 0 | 0 | 0 | 0 | 0 | 0 |
| CBM34 | 2 | 2 | 1 | 0 | 0 | 0 | 0 | 0 | 0 | 0 | 0 |
| CBM40 | 0 | 0 | 0 | 0 | 2 | 0 | 0 | 0 | 0 | 1 | 1 |
| CBM50 | 215 | 250 | 96 | 16 | 1 | 1 | 9 | 2 | 12 | 3 | 7 |
| CBM51 | 10 | 4 | 4 | 0 | 4 | 4 | 10 | 0 | 0 | 0 | 0 |
|  |  |  |  |  |  |  |  |  |  |  |  |
| **Dockerin** | 9 | 7 | 0 | 0 | 0 | 0 | 0 | 0 | 0 | 0 | 1 |
| **Cohesin** | 17 | 32 | 0 | 0 | 1 | 2 | 1 | 0 | 1 | 2 | 1 |

^a^ Number of different sequences in each mangrove sample.

MABA = mangrove BA; MARJ = mangrove RJ; Termite1 = *Nasutitermes termites* gut microbiome (Warnecke et al. 2007); Termite2 = *Trichonympha termites* gut microbiome; Panda1,2,5 = wild panda gut microbiome (Zhu et al. 2011); SoilL = soil microbiome from Colorado Plateau and Sonoran desert; Eupho, Meso, Omin = North Pacific Ocean (DeLong et al. 2006). All metagenomes are deposited in IMG/M database.
